# CRISPR/Cas9 Directed Mutagenesis of *OsGA20ox2* in High Yielding Basmati Rice (*Oryza sativa* L.) Line and Comparative Proteome Profiling of Unveiled Changes Triggered by Mutations

**DOI:** 10.3390/ijms21176170

**Published:** 2020-08-26

**Authors:** Gul Nawaz, Babar Usman, Neng Zhao, Yue Han, Zhihua Li, Xin Wang, Yaoguang Liu, Rongbai Li

**Affiliations:** 1 College of Agriculture, State Key Laboratory for Conservation and Utilization of Subtropical Agro-Bioresources, Guangxi University, Nanning 530004, China; gulnawazmalik@yahoo.com (G.N.); babarusman119@gmail.com (B.U.); nengzhao_gxu@163.com (N.Z.); hanyue0624@126.com (Y.H.); lizhihua-88@163.com (Z.L.); xinwang0112@126.com (X.W.); 2 State Key Laboratory for Conservation and Utilization of Subtropical Agricultural Bioresources, South China Agricultural University, Guangzhou 510642, China

**Keywords:** rice, gibberellins, plant height, CRISPR/Cas9, *OsGA20ox2*, proteomic analysis

## Abstract

In rice, semi-dwarfism is among the most required characteristics, as it facilitates better yields and offers lodging resistance. Here, semi-dwarf rice lines lacking any residual transgene-DNA and off-target effects were generated through CRISPR/Cas9-guided mutagenesis of the *OsGA20ox2* gene in a high yielding Basmati rice line, and the isobaric tags for relative and absolute quantification (iTRAQ) strategy was utilized to elucidate the proteomic changes in mutants. The results indicated the reduced gibberellins (GA_1_ and GA_4_) levels, plant height (28.72%), and flag leaf length, while all the other traits remained unchanged. The *OsGA20ox2* expression was highly suppressed, and the mutants exhibited decreased cell length, width, and restored their plant height by exogenous GA_3_ treatment. Comparative proteomics of the wild-type and homozygous mutant line (GXU43_9) showed an altered level of 588 proteins, 273 upregulated and 315 downregulated, respectively. The identified differentially expressed proteins (DEPs) were mainly enriched in the carbon metabolism and fixation, glycolysis/gluconeogenesis, photosynthesis, and oxidative phosphorylation pathways. The proteins (Q6AWY7, Q6AWY2, Q9FRG8, Q6EPP9, Q6AWX8) associated with growth-regulating factors (*GRF2*, *GRF7*, *GRF9*, *GRF10*, and *GRF11*) and GA (Q8RZ73, Q9AS97, Q69VG1, Q8LNJ6, Q0JH50, and Q5MQ85) were downregulated, while the abscisic stress-ripening protein 5 (*ASR5*) and abscisic acid receptor (*PYL5*) were upregulated in mutant lines. We integrated CRISPR/Cas9 with proteomic screening as the most reliable strategy for rapid assessment of the CRISPR experiments outcomes.

## 1. Introduction

Rice is a very important crop for the developing world and is considered a staple food for half of the world’s population [1,2,3,4]. Basmati rice has a unique specialty with good palatability, longer shelf life, easy digestibility, and good aroma [5]. The tall stature of traditional Basmati varieties is a big drawback as such varieties are easily lodged, which negatively affects the rice yield [6]. Plant height (PH) is an important character owing to critical roles in the plant architecture and adaptability to the environmental conditions that are directly linked with yield [7,8]. The number and length of the internodes determine the PH [9], which results in improved lodging resistance capacity and better biomass production due to the short stature [10]. Gibberellin (GA) is a plant growth hormone and many genes are vital for GA biosynthesis and signaling, which plays decisive roles in the metabolism of brassinosteroid and influences the stem length regulation in plants [11]. Mutations in components participating in GA signaling and metabolism pathways exhibit notable dwarf or semi-dwarf plant phenotype [12]. The tall phenotype is controlled by the *SD1* (semi dwarf1) allele while its recessive allele (*sd1*) controls the semi-dwarf phenotype [13,14]. The *OsGA20ox2* has 3 exons and 2 introns, a total of 389 amino acids are encoded by this gene and GA20 oxidase2 is a major enzyme for GA synthesis [13]. Mostly, GAs, consist of large groups of diterpenoid carboxylic acids such as GA1 and GA4, and display a vital role in the development and growth of plants [15,16]. *OsGA20ox2* is mainly expressed in the stem, and mutations in *OsGA20ox2* resulted in the production of a moderate amount of GA, and its mutants with loss-of-function exhibit a semi-dwarf phenotype [10,17,18]. The miracle rice cultivar IR8 has a short plant height because of the *SD1* gene mutation and enabled extraordinary yield increases and facilitated the prevention of food shortages [13,18,19]. The *OsGA20ox2* mutants have no negative effects on the morphological and yield-related traits and extensive efforts have been made by the research community for achieving the semi-dwarf phenotype in rice through molecular breeding and PH can be restored in mutants similar to that of WT (wild type) plants by exogenous GA_3_ treatment [20,21,22].

The targeted and effective gene mutations have been successfully achieved by the CRISPR/Cas9 (clustered regularly interspaced short palindromic repeats/CRISPR-associated protein 9) system in rice for improving the existing varieties and to develop new mutant lines by targeting one or multiple genes [23,24,25]. CRISPR/Cas9 is a tremendous technique for precise and targeted editing of the genome of plants and animals. ZFNs (zinc finger nucleases) and TALEN (transcriptional activator-like effector nuclease) genome editing techniques were established before the CRISPR/Cas9, but because of the simplicity and flexibility, rapidness, multiplexing capacity, high efficiency, and mutation frequency, this system gained worldwide popularity and is widely accepted by researchers [26,27]. The CRISPR/Cas9 technique has been successfully applied in Arabidopsis [28,29], rice [28,30,31,32], maize [33], wheat [31,34], sorghum [28], soybean [35,36,37], and tomato [38], and the resulted mutations are transmitted to the next generations according to the classical inheritance principles [39].

The development of the proteomics over the last few decades has greatly contributed in omics and is now accepted widely to study various species [40,41,42,43,44]. To comprehend the effects of mutations on the genome of plants, whole genome-wide profiling of proteins or transcripts is an efficient way to investigate the distinct variations in diverse biological and molecular processes [45]. The dramatic improvement in the methods used in molecular biology, extensive profiling of transcripts, and iTRAQ (isobaric tags for relative and absolute quantification)-based proteomic analysis can provide insights into a universal view of genes and protein expression patterns and can be helpful to understand the potential molecular mechanism behind the mutagenesis [46,47]. The iTRAQ has been applied successfully in plant species to recognize the diverse biological processes, including Arabidopsis [48], rice [49,50], wheat [51], and maize [52]. It is an effective and high-throughput approach with high sensitivity and accuracy, multiplexing capacity, and repeatability. However, this tremendous method has not yet been utilized to investigate which mechanisms are underlying the semi-dwarf phenotype. In our study, we have exploited the CRISPR/Cas9 technology to knock out the *OsGA20ox2* gene in Basmati rice, and earmark and delicate mutations in the *OsGA20ox2* gene and homozygous T_1_ semi-dwarf mutant lines were achieved effectively, with significantly reduced PH and GA content without disturbing other agronomic characters. To further understand the functional roles of the *OsGA20ox2* gene, comparative iTRAQ analysis was performed to elucidate the effects of mutations on the protein level. Our results provide new hints in understanding *OsGA20ox2* functions and suggest that the proteomic screening is a reliable tool for assessment of CRISPR experiments. By this tactic, our aim was to infer the mechanisms that are more closely associated with rice dwarfism.

## 2. Results

### 2.1. Editing of OsGA20ox2 Gene and Identification of Transgene-Free Plants

**Figure 1:**
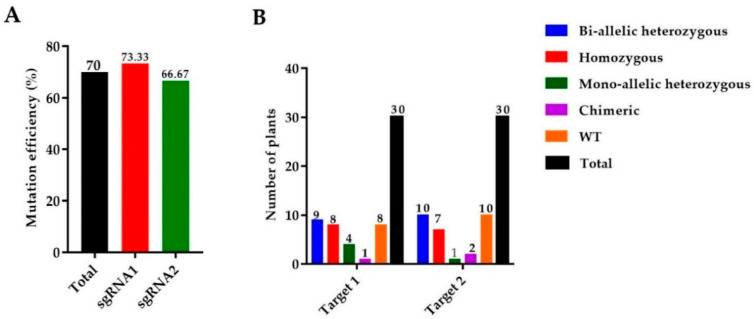

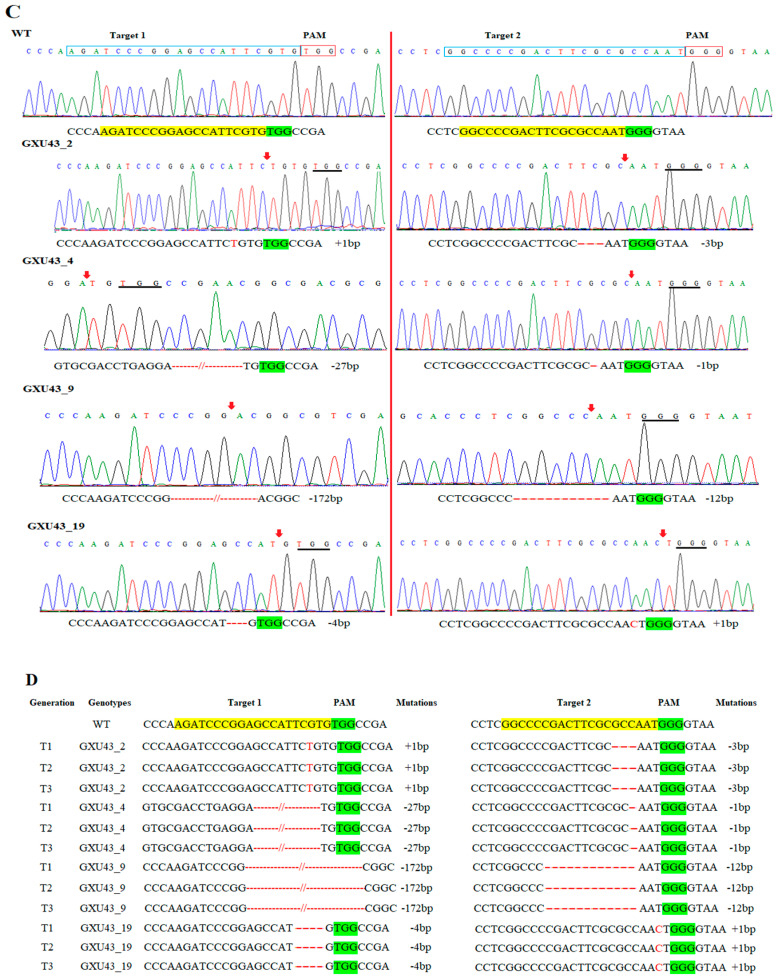
Identification of mutations generated by the clustered regularly interspaced short palindromic repeats/CRISPR-associated protein 9 (CRISPR/Cas9) system. (**A**) The mutation efficiency of sgRNAs (single guided RNAs), (**B**) The mutation rate in T_0_ (Transgenic) generation, (**C**) sequencing chromatograms of WT (wild type) and homozygous mutant lines for Target 1 and Target 2, and (**D**) the alignment of sequences for T_1_, T_2_, and T_3_ generations, respectively. The target sequence is painted in yellow, while the PAM (protospacer adjacent motif) is in the green background, and insertions/deletions are represented by red hyphens and letters. The analysis was carried out in three replications for each line.

We used 165 calli for the *A. tumefaciens* transformation and a total of 30 rice seedlings were achieved. Mutant lines were screened by using hygromycin phosphotransferase (HPT-F/R) primers, and the final product was amplified and confirmed in mutants (Supplementary file 1, Table S1, Figure S1A). A high rate of mutation was observed in both targets of the *OsGA20ox2* gene, with a total mutation efficiency of 70% (73.33% for T_1_ and 66.33% for T_2_). Among 30 plants, there were 8 (26.67%) homozygous, 9 (30%) biallelic (heterozygous), 4 (13.33%) heterozygous (mono-allelic), and 1 (3.33%) chimeric. A total of 30 mutant lines were obtained for T_2_, including 7 (23.33%) homozygous, 10 (33.33%) biallelic (heterozygous), 1 (3.33%) heterozygous (mono-allelic), and 2 (6.67%) were chimeric (Figure 1A,B). Mutated alleles were amplified from the genomic DNA of T_0_ mutant lines and the sequencing chromatograms with overlapping traces were decoded (Figure 1C).

We selected four transgene-DNA-free (T-DNA-free) homozygous lines (GXU43_2, GXU43_4, GXU43_9, and GXU43_19) for agronomic traits evaluation. GXU43_2 showed homozygous mutations with 1 bp insertion and 3 bp deletion, the mutant GXU43_4 showed 27 bp and 1 bp deletions, the homozygous mutant GXU43_9 revealed 172 bp and 12 bp deletions, and finally, the homozygous mutant line GXU43_19 resulted in 4 bp deletion and 1 bp insertion on the first and second target sites, respectively. The sequencing analysis of these homozygous mutants showed that the mutations were stable and inheritable in T_1_, T_2_, and subsequent T_3_ generations (Figure 1D). The plant numbers and the corresponding mutations in both target sites are mentioned in Supplementary file 1, Table S2. The thirty plants were selected, and the DNA was amplified for 5 loci of each target site with the highly ranked off-target potential, and no secondary off-target mutations were detected in sequencing results (Supplementary file 1, Table S3).

### 2.2. T-DNA-Free Mutants and Segregation Ratio in the T_1_ Generation

Homozygous mutants of T_0_ and T_1_ were grown and 50 plants were evaluated to analyze the transformation patterns and thus to detect the exogenous DNA in the mutant lines. The plants were believed to be T-DNA-free if they failed to amplify against HPT and Cas9-specific primers (Supplementary file 1, Table S1). The results showed that 30 plants were amplified to the Cas9 vector sequence and 20 lines showed no amplification, and therefore, were considered as T-DNA-free. The frequency of such plants was recorded at 40% (Supplementary File 1, Figure S1B). The heterozygous (mono and biallelic) T_1_ plants of GXU43_8 were segregated according to Mendelian inheritance (1:2:1). All the T_1_ plants obtained from T_0_ homozygous plants (GXU43_2, GXU43_4, GXU43_9, and GXU43_19) also showed homozygosity for the same mutations, which indicated the stable transmission of mutations to the subsequent generations (Supplementary File 1, Table S4).

### 2.3. Endogenous GA Content and PH in T_1_, T_2_, and T_3_ Generations

The mutant lines exhibited decreased PH (plant height) and GA content (GA_1_, GA_4_) as compared with WT plants. The mutant line GXU43_9 showed a minimum PH of 114.77, 115.55, and 113.98 cm in T_1_, T_2_, and T_3_ generations respectively, and the GA_1_ and GA_4_ content (0.95 and 0.84 in T_1_, 0.91 and 0.81 in T_2_, and 0.88 and 0.87 μg/kg fresh weight (FW) in T_3_ generation, respectively), while WT showed maximum plant height (161.31 cm) and gibberellins (1.66 and 1.54 μg/kg FW) content. The results revealed a significant and positive correlation among endogenous GA content and PH of WT and transgenic plants (Table 1).

### 2.4. Performance of Agronomic and Quality Traits

**Figure 2:**
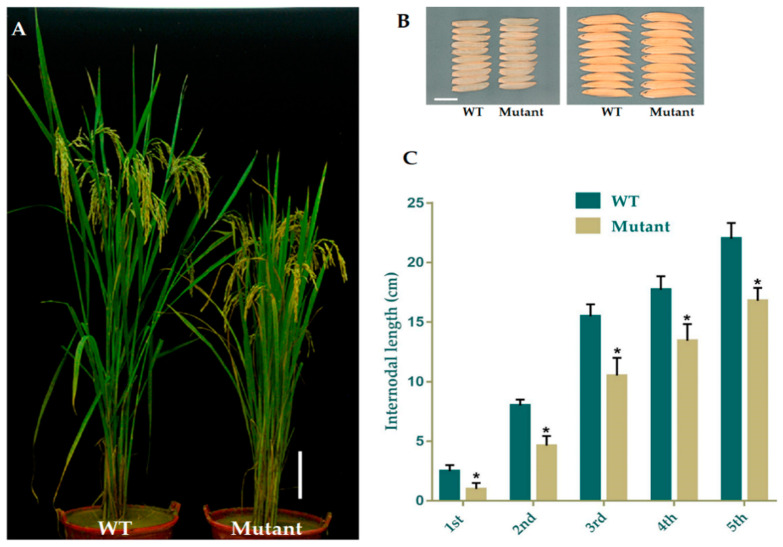
Phenotypic appearance of *OsGA20ox2* in GXU43_9 and WT. (**A**) Plant height of mutant lines and WT after the heading stage. Bar = 15 cm. (**B**) Grain phenotype of mutant line and WT; Bar = 5 mm. (**C**) The lengths of internodes in mutant line and WT. The “*” denotes the significant difference at *p* < 0.01. Values are means ± standard deviation (SD) (*n* = 10 plants).

The data for the major agronomic traits of mutant and WT plants were recorded at 120 days after growing. The mean results for morphological traits expressed a significant difference amongst WT and mutant plants in PH (Figure 2A) and FLL (flag leaf length), while semi-dwarf lines showed slightly shorter PL (panicle length), with no difference in PN (panicle numbers), FLW (flag leaf width), GNPP (grain number per panicle), SSR (seed setting rate), and GW(1000-grain weight), YPP (yield per plant), GL (grain length), and GWD (grain width). We selected the four homozygous mutant lines GXU43_2, GXU43_4, GXU43_9, and GXU43_19, and the results of data for three consecutive generations revealed that the PH and FLL of mutants were significantly decreased (Table 2). However, grain appearance and shape were not altered (Figure 2B). Mutant lines showed shortened internodal length than WT plants (Figure 2C).

### 2.5. Effect of Exogenous GA_3_

**Figure 3:**
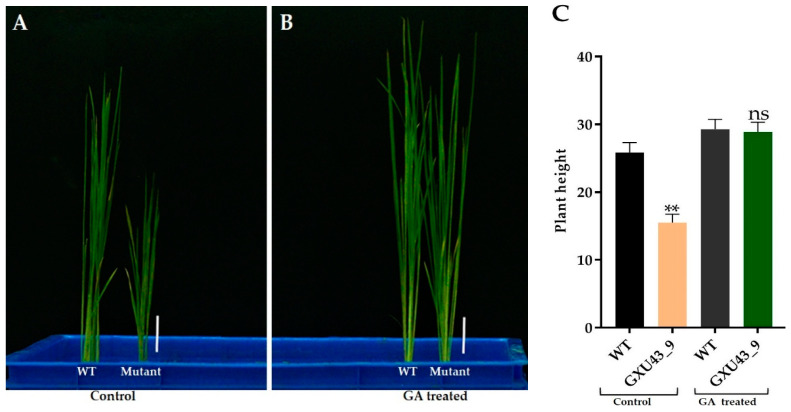
The seedling phenotype of the homozygous mutant GXU43_9 and WT. (**A**) Seedlings phenotype without GA, (**B**) Seedlings treated with GA, and (**C**) PH (plant height)of GA3-treated and control (*n* = 15), Bars = 3 cm. Data are mean ± SD. “**” and “^ns^” represent a significant and non-significant difference respectively at *p* ≤ 0.01.

We applied 10 μM GA_3_ to analyze the response of mutant and WT plants at the seedling stage. The data for plant height was recorded after 25 days. The mutant plants responded to exogenous GA_3_ significantly and restored the PH identically to that of the WT (Figure 3A,B). The homozygous mutant line GXU43_9 exhibited the lowest PH (15.52 cm) in controlled conditions as compared to the PH of wild type plants (25.85 cm), which was significantly higher than the mutant plants. Under the GA_3_ application, the T_1_ mutant line GXU43_9 restored the PH (28.95 cm), nearly equal to that of WT plants (29.24 cm). These results clearly show that the GA_3_ application promoted the growth of WT and mutant plants (Figure 3C).

### 2.6. Section Analysis of Culm Cells

**Figure 4:**
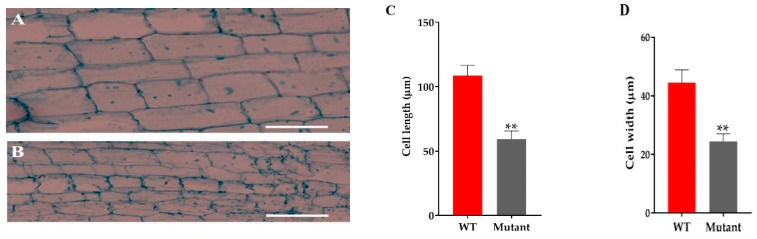
The microscopic analysis of culm cells of WT and mutant line GXU43_9. (**A**) WT second intermodal longitudinal length of the cell and (**B**) mutant plant GXU43_9, respectively. (**C**,**D**) Quantitative measurement of the length and width of the cells of mutant line (GXU43_9) and wild-type plants (*n* = 15), Bars = 100 μm. Data are the mean ± SD, “**” shows the significant difference at *p* ≤ 0.01.

The microscopic analysis of the culm cell of mutant line GXU43_9 showed irregularly shaped cells with thin walls, significantly smaller in size, and an increase in cell layers was observed as compared to WT (Figure 4A,B). From these results, we can conclude that the development of the stalk was affected in GXU43_9. The length and width of the cells in mutants was significantly smaller as compared to WT (Figure 4C,D).

### 2.7. Proteomics Analysis

**Figure 5:**
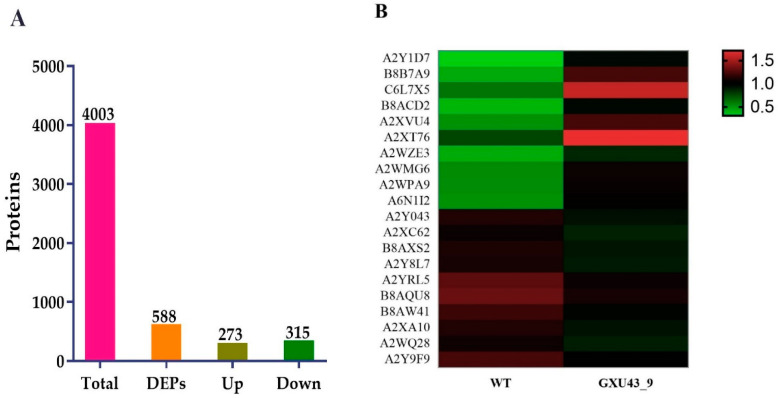
Proteomic analysis information of the CRISPR mutant GXU43_9 and its WT. (**A**) Upregulated and downregulated differentially expressed proteins (DEPs), and (**B**) Heatmap of the top twenty DEPs. Red color denotes the higher while the green color represents a lower level of expression.

A total of 267,114 spectra was generated, and after the analysis of these spectra, we identified 68,489 known spectra with 24,230 peptides and 4003 proteins, respectively (Supplementary Figure S2A). The protein mass distribution is represented in Supplementary Figure S2B. The proteins having 20–40 kDa represented more protein numbers, and proteins with 141–150 kDa showed less protein numbers. The number of peptides identified in the proteins is shown in Supplementary Figure S2C. The information about the distribution of peptide length and protein sequence coverage is shown in Supplementary Figure S2D,E. After implementing the analysis with a minimum fold change (FC) of ≥1.2 and *p*-value adjusted to ≤0.05, a total of 588 DEPs (273 upregulated and 315 downregulated) were obtained (Figure 5A). The detailed information of all the proteins identified in this analysis is given in Supplementary file 2.

The top 30 up- and down-regulated proteins were selected based on the highest log2 FC value, of which the top 10 upregulated DEPs were Metallothionein-like protein 3B (A2Y1D7), Uncharacterized protein (B8B7A9), Ethylene response factor (C6L7X5), Uncharacterized protein (B8ACD2), Uncharacterized protein (A2XVU4), Abscisic stress-ripening protein 5 (Q53JF7), Uncharacterized protein (A2WZE3), Salt stress root protein (A2WMG6), Peroxidase (A2WPA9), and ATP-dependent clp protease proteolytic subunit (A6N1I2). While the Peroxidase (A2Y043), Uncharacterized protein (A2XC62), Uncharacterized protein (B8AXS2), Peptidase A1 domain-containing protein (A2Y8L7), Growth-regulating factor 9 (Q9FRG8), MFS domain-containing protein (B8AQU8), NAD(P)-bd_dom domain-containing protein (B8AW41), Uncharacterized protein (A2XA10), Ent-copalyl diphosphate synthase 2 (Q5MQ85), and ATP-dependent 6-phosphofructokinase (A2Y9F9) were downregulated in the mutant line (Figure 5B).

We further searched the DEPs related to GA and plant growth. We found that five DEPs (Q6AWY7, Q6AWY2, Q9FRG8, Q6EPP9, and Q6AWX8) related to growth-regulating factors (*GRF2*, *GRF7*, *GRF9*, *GRF10*, and *GRF11*) were downregulated in this report. The DEPs related to gibberellin response modulator-like proteins (Q8RZ73 and Q9AS97), Chitin-inducible gibberellin-responsive proteins (Q69VG1 and Q339D4), Putative gibberellin oxidase (Q8LNJ6), Ent-copalyl diphosphate synthase 2 (Q5MQ85), gibberellin 20 oxidase 2 (Q0JH50), ATP synthase subunit beta chloroplastic (J7EYN3), and fructose-1,6-bisphosphatase, chloroplastic (A2XEX2) were also downregulated in mutant lines. However, the DEPs (Abscisic stress-ripening protein 5) and Q6I5C3 (Abscisic acid receptor) were upregulated in mutant plants (Table 3).

### 2.8. DEPs Functional Networks and Hub-Protein Analysis

**Figure 6:**
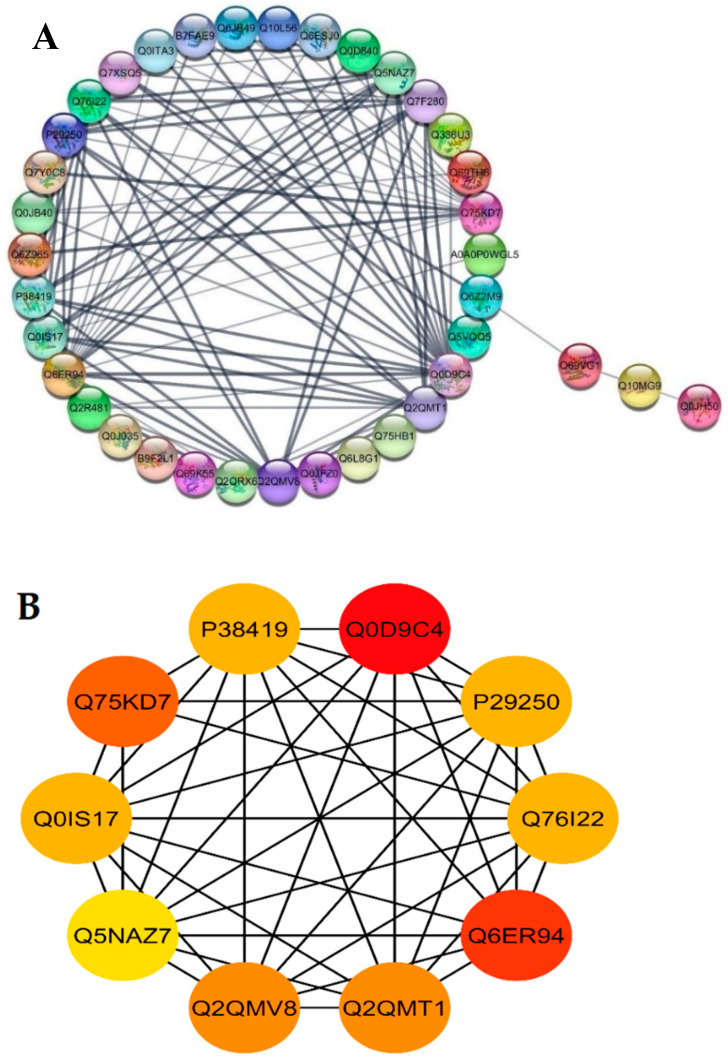
Functional networks of DEPs and hub-protein analysis of WT and mutant line GXU43_9. (**A**) STRING software predicted proteins’ associations. The nodes represent differentially expressed proteins, while the edge denotes the interaction relationship between the nodes. (**B**) The top hub-proteins of WT and GXU43_9 comparison. The higher co-expression is denoted by red color.

The STRING database was used for retrieving the protein interaction networks; for this purpose, the confidence (score) cutoff was adjusted to 50 with 30 additional interactors. The nodes represent the proteins, and protein-protein interaction (PPI) modes are shown by the lines among the nodes. The analysis of network revealed a higher co-expression between catalase isozyme B (Q0D9C4), 2-Cys peroxiredoxin BAS1 (Q6ER94), protein coleoptile photomorphogenesis 2 (Q75KD7), adenine phosphoribosyltransferase 1, putative, expressed (Q2QMV8), adenine phosphoribosyltransferase 1, putative (Q2QMT1), linoleate 9S-lipoxygenase 2 (P29250), lipoxygenase 7, chloroplastic (P38419), lipoxygenase (Q0IS17), linoleate 9S-lipoxygenase 1 (Q76I22), probable indole-3-acetic acid-amido synthetase (Q5NAZ7), isocitrate dehydrogenase (NADP) (Q7F280), and phloem sap 13 kDa protein 1 (Q0D840), which showed a higher interaction score of ≥5 (Figure 6A).

Highly connected to 10 hub proteins with a higher degree of connectivity by the STRING database, the following were selected as candidate hub proteins: the adenine phosphoribosyltransferase 1, putative, expressed (Q2QMV8), adenine phosphoribosyltransferase 1 (Q2QMT1), linoleate 9S-lipoxygenase 2 (P29250), lipoxygenase 7, chloroplastic (P38419), lipoxygenase (Q0IS17), linoleate 9S-lipoxygenase 1 (Q76I22), probable indole-3-acetic acid-amido synthetase (Q5NAZ7), and 2-Cys peroxiredoxin BAS1, chloroplastic (Q6ER94) (Figure 6B).

### 2.9. Gene Ontology (GO) and Pathway Enrichment Analysis

**Figure 7:**
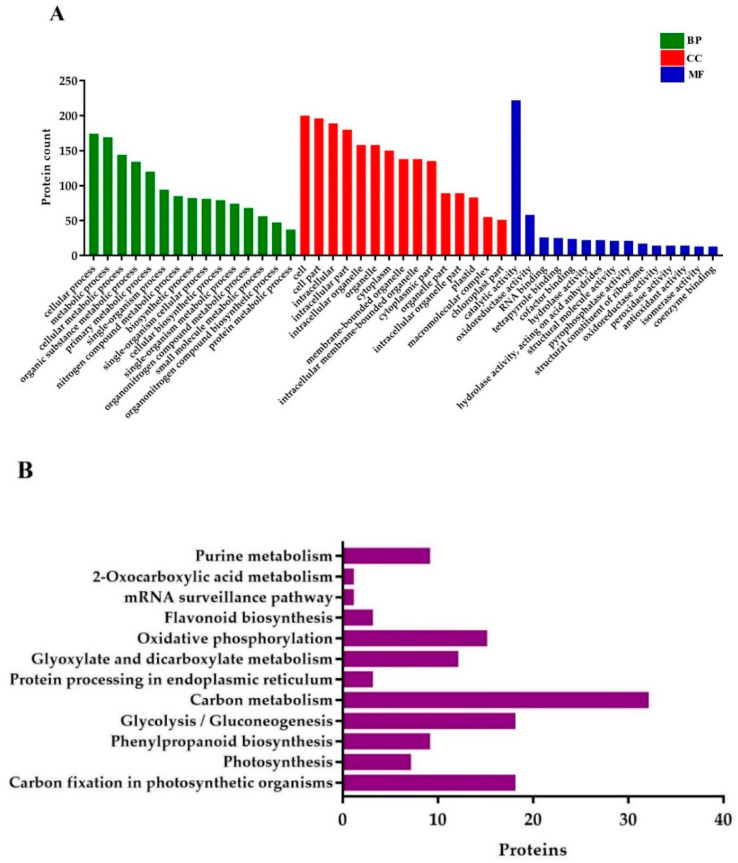
Gene ontology (GO) and KEGG (Kyoto Encyclopedia of Genes and Genomes) pathway enrichment analysis of DEPs. (**A**) GO annotations of the DEPs, and (**B**) the histogram of KEGG pathway enrichment, with the bar showing the number of proteins. BP; biological processes, CC; cellular components, and MF; molecular functions.

GO annotations for DEPs related to BP (biological processes) were associated with the cellular process, metabolic process, cellular metabolic process, organic substance metabolic process, primary metabolic process, single-organism process, nitrogen compound metabolic process, biosynthetic process, single-organism cellular process, and cellular biosynthetic process. Proteins conferring CC (cellular components) were mainly involved in cell, cell part, intracellular (intracellular part, organelle, and membrane-bounded), organelle (primary and membrane-bounded), cytoplasm, and cytoplasmic part. Finally, for the MF (molecular functions) perspective, the DEPs took part in catalytic activity, oxidoreductase activity, RNA binding, tetrapyrrole binding, hydrolase activity (primary and cofactor binding), acting on acid anhydrides in phosphorus-containing anhydrides, acting on acid anhydrides structural molecule activity, pyrophosphatase activity, and structural constituent of ribosome (Figure 7A).

KEGG (Kyoto Encyclopedia of Genes and Genomes) pathway analysis uncovered that the DEPs were mostly enriched in carbon fixation in photosynthetic organisms, photosynthesis, phenylpropanoid biosynthesis, glycolysis/gluconeogenesis, protein processing in the endoplasmic reticulum, carbon metabolism, glyoxylate and dicarboxylate metabolism, oxidative phosphorylation, flavonoid biosynthesis, mRNA surveillance pathway, and 2-Oxocarboxylic acid metabolism (Figure 7B).

### 2.10. RT-qPCR Analysis of Target Gene and Proteomic Data Validation

**Figure 8:**
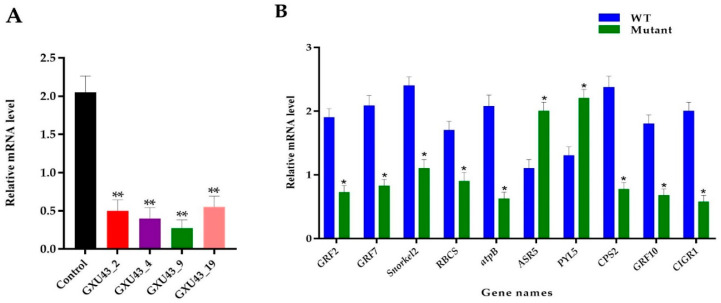
Real-time quantitative PCR assessment and validation of proteomics data. (**A**) Expression analysis of *OsGA20ox2*, in WT and T_1_ mutant lines, (**B**) RT qPCR validation of the ten DEPs responsive genes. The SD is shown with error bars, “*” represents the significant differences at *p* ≤ 0.05 and “**” represents the significant differences at *p* ≤ 0.01 respectively; *n* = 3.

The RT-qPCR was used to assess the relative expression of the *OsGA20ox2* gene in mutants and WT plants. To normalize the expression, the Rice *Actin* gene was used as a reference between the samples, and the expression of *OsGA20ox2* was significantly suppressed in all mutant lines (*p* < 0.01, Figure 8A). Ten genes associated with DEPs were selected for the validation of the proteomic data, and the qRT-PCR assay was performed for independent samples of WT and mutant lines. In total, eight key genes encoding downregulated DEPs, including *GRF2*, *GRF7*, ethylene response factor gene (*Snorkel2*), ribulose bisphosphate carboxylase small-chain gene (*RBCS*), ATP synthase subunit beta (*atpB*), Ent-copalyl diphosphate synthase 2 gene (*CPS2*), *GRF10*, and Chitin-inducible gibberellin-responsive protein1 (*CIGR1*)*,* and two genes encoding upregulated DEPs, including abscisic stress-ripening protein 5 gene (*ASR5*) and Abscisic acid receptor (*PYL5*), were chosen. The level of expression of selected genes was consistent with the proteomic analysis (Figure 8B). Primers used for RT-qPCR are given in Supplementary file 1, Table S1.

## 3. Discussion

The rise of modern molecular breeding technologies has provided fast and efficient means for plant breeding, which can directly modify desirable traits without changing and affecting other traits. CRISPR/Cas9 is determined by the easily and cheaply modified sgRNA (Single guided RNA) followed by a short PAM (protospacer adjacent motif) sequence to induce mutations with high accuracy and reduced probability of off-targets [53,54,55]. Crop improvement by using gene editing suggests decent prospects to generate mutants with preferred traits. However, there are very few examples of the utilization of SSNs (sequence-specific nucleases) for the generation of novel genotypes with desired plant type. The CRISPR/Cas9 technique has been extensively used in many species for the targeted mutagenesis because of its high efficiency. In this study, we have successfully used this system to edit the *OsGA20ox2* gene with a higher mutation efficiency. Mutants are very important for genetic research and crop breeding. However, the assimilation of desired genes into elite breeding varieties is still a challenging task.

Breeding productivity is restricted by the continuous selection process, which is the main drawback of conventional breeding [56,57]. The natural selection events occur at random and result in unguided mutagenesis that brings a little frequency of, mutational events at target loci [57]. On the other hand, CRISPR technology has a great potential to produce mutants by the targeted mutagenesis in pre-decided locus [49]. We employed the iTRAQ strategy to confirm the effects of CRISPR-based gene editing at the whole proteome level.

The CRISPR/Cas9 vector has two key functional parts, including a gRNA and Cas9 expression cassettes, driven by RNA polymerase III and 35S/ubiquitin promoters, respectively. Because the Pol III has a limited transcriptional capacity, previously, researchers used U3/U6 promoters from rice, which have specific transcriptional sites with nucleotides A and G, respectively [58,59]. We used U6 (OsU6a, OsU6) promoters to construct the expression cassette, the target sequences were selected with 5′-GN(19)NGG and, 5′-AN(19)NGG, respectively. Two sgRNAs were ligated successfully in the U6 promoters-driven expression cassette for targeting *OsGA20ox2*. In the present work, the CRISPR/Cas9 tool was used for editing the *OsGA20ox2* gene in the Basmati rice line. The heterozygous and homozygous mutation events were found frequently, however, the chimeric mutations were rare. The homozygous and compound heterozygous mutations are also found frequently in previous studies [60,61]. The homozygous mutant plants achieved in T_0_ generation showed stable and inheritable mutations to the subsequent generations. In previously reported studies the homozygous mutations have also been achieved in T_0_ generation [61]. In this study, the mean mutation efficiency obtained was 70%, and the mutant lines were obtained without any off-target mutations. The previously reported work also suggests that the Cas9 rarely tempts off-target mutations in rice [62,63], and we screened the T-DNA-free lines at a frequency of 40%. The segregation and predicted inheritance have great importance in molecular breeding. In our study, we found inheritable and highly stable mutations induced by Cas9. In segregation analysis, the homozygous mutations were transmitted stably, showing no inversions or new mutations in T_1_ according to the Mendelian principle. Bi-allelic and heterozygous mutations were segregated at a 1:2:1 ratio, while the chimeric mutations were found to be unpredicted. We can conclude from the results that the new mutations are not induced by Cas9, while further experiments are needed to uncover the unpredictable segregations of chimeric mutations [61,64].

The GA biosynthesis and deactivation genes confer endogenous GA levels and response to GA metabolism [12,65,66,67,68,69]. Semi-dwarfism is a very important trait and rice varieties with this feature brought about significant improvements in grain production. GA is considered as the main plant growth-promoting hormone for triggering stem elongation, which experiences dynamic cell division for controlling plant development and growth [70]. The expression level of *OsGA20ox2* was significantly reduced and mutant lines exhibited semi-dwarf PH at seedlings and mature stages, with significantly lowered GA content. Studies have revealed that GA_3_ promotes stem and sheath elongation and synthesis of many proteins related to plant growth [71,72,73]. The mutant plants restored their PH with exogenous GA_3_ application. The previous studies also showed that actual height similar to WT can be restored through GA_3_ treatment [74]. *OsGA20ox2* CRISPR mutants also showed increased number and layers of cells, but decreased cell length and width, which may be the main cause of shortened PH. Dwarf plants usually show compact cell size and this results in affected cell expansion [75].

A total of 588 DEPs were identified in GXU43_9 versus the WT comparison, and among them, 273 were upregulated and 315 were downregulated, respectively. Some highly expressed proteins controlling PH were also identified in mutant lines. The DEPs including Q6AWY7, Q6AWY2, Q9FRG8, Q6EPP9, and Q6AWX8 (*GRF2*, *7*, *9*, *10*, *11*) were downregulated in mutant lines. In rice, the GRF family of TFs (transcription factors) has been previously identified [76], which are known as plant-specific TFs that were firstly known for their developmental role in leaf and stem growth [77]. *OsGRF1* is a GA-induced gene in intercalary meristem internodes, and was the first GRF to be recognized in rice [78]. Some GRFs’ expression level in Arabidopsis and rice is usually high in the tissues growing actively; besides, plants showed increased expression levels for several GRFs (*OsGRFs* 1, 2, … 12) after exogenous GA_3_ treatment, whereas *OsGRF9* showed decreased expression [79,80,81]. *OsGRF6* shows higher expression in developing inflorescences, which showed that GRFs are also involved in organ growth and development related to floral parts [82]. The gibberellin response modulator-like proteins (Q8RZ73 and Q9AS97), Chitin-inducible gibberellin-responsive proteins (Q69VG1 and Q339D4), Putative gibberellin oxidase (Q8LNJ6 and), Ent-copalyl diphosphate synthase 2 (Q5MQ85), gibberellin 20 oxidase 2 (Q0JH50), ATP synthase subunit beta chloroplastic (J7EYN3), fructose-1,6-bisphosphatase, chloroplastic (A2XEX2), and Ethylene response factor (C6L7X5) were also downregulated in mutant lines. Chitin-inducible GA responsive proteins (CIGR1 and CIGR2), inducible by the potent elicitor *N*-acetylchitooligosaccharide (GN), are fast induced by exogenous GA. The expression of proteins is reliant on the quantity and biological activity of GA, showing that the expression of these genes is mediated by GA [83]. Ent-copalyl-diphosphate act in the biosynthesis of defensive phytoalexin and GA phytohormone, which generally functions in GA biosynthesis, as mutations in the gene controlling this protein result in weakened growth [84]. Gibberellin 20 oxidase 2 is a major oxidase enzyme for the GA biosynthesis that is responsible for the catalyzation process which converts GA53 to GA20 through an oxidation reaction at C-20 of the GA skeleton and takes part in the internodal elongation [19].

Ribulose bisphosphate carboxylase (large and small chain) was downregulated in the mutant line and it was reported that RuBisco plays a significant role in the accumulation of chlorophyll and photosynthesis, and its overexpression leads to increased photosynthetic activity to attain growth [85]. The TPSs (terpene synthases) family is responsible for terpene molecules in plants, which play a key role in primary metabolism. In bryophyte, only a single TPS gene, *CPS* (*copalyl synthase*), is a precursor of GA, which encodes a bi-functional enzyme-producing ent-kaurene [86]. Loss-of-function mutants of SOTs (sulfotransferases) and TPST (tyrosylprotein SOTs) showed various abnormal characteristics due to peptides or proteins’ sulfurization related to growth and development [87,88]. The TPST activity was previously observed in rice during microsomal membrane preparations [89]. The ethylene response factor (product of *SNORKEL2*) triggers notable internode elongation via gibberellin [90]. However, the DEPs, A2WPN7 (Salt stress-induced protein), A2WMG6 (Salt stress root protein), Q53JF7 (Abscisic stress-ripening protein 5), and Q6I5C3 (Abscisic acid receptor), were upregulated in mutant line GXU43_9 as compared to WT. Abscisic stress-ripening protein 5 was engaged in the GA signaling pathway and plant growth regulation in the region extending to basal leaf sheaths. The expression regulation of various genes is also carried out by ASR5 that contributes to cell protection against aluminum stress in rice plants [91]. In rice, the overexpression of *PYL5* enhances abiotic stress tolerance and inhibits growth through gene expression modulation [92].

GO annotations of DEPs revealed that most of the proteins related to BP were associated with cellular and organic substance metabolic processes, the single-organism process, nitrogen compound metabolic process, and cellular biosynthetic process. Proteins conferring CC were associated with cell, cell part, intracellular (intracellular part, organelle, and membrane-bounded), organelle (primary and membrane-bounded), cytoplasm, and cytoplasmic part. Finally, for the MF perspective, the DEPs were mainly involved in catalytic activity, oxidoreductase activity, RNA binding, tetrapyrrole binding, cofactor binding hydrolase activity, hydrolase activity, pyrophosphatase activity, and structural constituent of ribosome. KEGG pathway analysis showed that the DEPs were enriched in carbon fixation in photosynthetic organisms, photosynthesis, and glycolysis/gluconeogenesis. In network analysis of hub-proteins, we found that most of the DEPs, including linoleate 9S-lipoxygenase 2, lipoxygenase 7, chloroplastic, lipoxygenase, and linoleate 9S-lipoxygenase 1, were related to metabolic processes that may be engaged in various diverse aspects associated with plant physiology including growth and development [93,94]. In the present study, the proteins related to plant growth and GA were downregulated, implying that they may be responsible for a series of biochemical and physiological changes related to plant growth and ultimately affecting plant height. In summary, the plant height reduction through the modification of *OsGA20ox2* expression levels in rice by affecting the biosynthesis of GA is potentially of great agronomic interest. This study showed that plant characteristics can be improved through genetic mutations. In this study, the successfully developed semi-dwarf mutant rice lines can be exploited for future breeding programs. Further studies regarding the cell signaling mechanisms owing to genome manipulations are warranted.

## 4. Material and Methods

### 4.1. Material Used and Field Conditions

Seeds of Basmati rice variety (VP-1643) were provided by the Wild Rice Group of Guangxi University (GXU) and plants were propagated in the experimental area of GXU, China, and at the Farm of Divisional Headquarters, Sanya Hainan, China, in the normal growing season and maintained consistently. The pYL CRISPR/Cas9Pubi-*H* vector (Supplementary file 1, Figure S3) and the promoters (OsU6a and OsU6b) (Supplementary file 1, Figure S4A–C) were used to construct plasmid. This expression vector possesses the HPT selectable marker with a sequence bordering *BsaI* sites for sgRNA expression cassette insertion (Supplementary file 1, Figure S5A) [95].

### 4.2. SgRNAs Selection, Vector Construction, and Transformation

The target sequences were selected after the confirmation of (N)_20_ GG or G(N)_20_ GG template in coding regions of the *OsGA20ox2* gene by employing the online website CRISPR-GE (http://skl.scau.edu.cn/) [96], with higher targeting specificity (Supplementary file 1, Table S5; Figure S5B). The two targets for *OsGA20ox2* were selected in the exon region from 128 to 147 bp and 541 to 560 bp, respectively. The structures of all sgRNA’s were developed by using an online tool, CRISPR-P (http://crispr.hzau.edu.cn/cgi-bin/CRISPR2/CRISPR) (Supplementary file 1, Figure S6). The overlapping PCR reaction was performed to construct the expression cassette [95] and is represented in Supplementary Figure S7, and the primers used are mentioned in Supplementary Table S1. The amplified product was purified by using TaKaRa MiniBEST Purification Kit Ver.4.0. The transformation of expression cassette to competent cells of *E. coli* DH5-alpha was performed according to a previously established method [97]. Primers SP-L1 and SP-R were used to assess the correct size of amplified products and sequenced directly (Supplementary file 1, Table S1). The sequences of target sites were confirmed in a constructed expression cassette. The order of U6 promoter-driven sgRNA cassettes was as follows: LacZ–OsU6a–T_1_–OsU6b–T_2_ (Supplementary file 1, Figure S8A). The sizes of sgRNA cassettes after amplification were as follows: OsU6a–sgRNA1: 629 bp, OsU6b–sgRNA: 515 bp (Supplementary file 1, Figure S8B). After transforming the expression vector into competent cells of DH5α the PCR amplification was performed to detect positive colonies (Supplementary file 1, Figure S8C). The sequence containing the target region of *OsGA20ox2* in WT was amplified (Supplementary file 1, Figure S8D). Sequencing results successfully confirmed the targets assembly in the vector (Supplementary file 1, Figure S8E). The CRISPR/Cas9 binary vector was successfully constructed, which was considered suitable for rice transformation and target gene editing.

The transformation of embryonic calli was accomplished by the Agrobacterium tumefaciens-mediated co-cultivation method, as previously established by Hiei et al. [98].

### 4.3. Genotyping, Off-Target Analysis, and Identification of Transgene-Free Plants

The DNA extracted by the CTAB (cetyl trimethylammonium bromide) method [99] and target-specific primers (*SD1T1* F/R and *SD1T2* F/R) were designed for the amplification of both target sites of *OsGA20ox2* gene (Supplementary file 1, Table S1). Sequences were decoded with DSDecode (http://skl.scau.edu.cn/dsdecode/) [100]. The CRISPR-GE online tool (http://skl.scau.edu.cn/) was accessed for the identification of the off-target sites (Supplementary file 1, Table S3) for the target regions, and five putative off-targets having ≥2 nucleotide mismatches for each target site were tested. The primers were designed, and PCR products were sequenced directly (Supplementary file 1, Table S6). The genomic DNA in T_1_ and T_2_ (T-DNA-free and T-DNA) was extracted for genotyping and to study the inheritance patterns. The screening of T-DNA-free plants was performed by Cas9-F/Cas9-R and HPT-F/HPT-R primers in the T_1_ generation. Those plants regarded as T-DNA-free which lack both HPT and Cas9 simultaneously. The T-DNA-free homozygous mutant plants were further analyzed to study different agronomic and biochemical parameters. The mutations transmission patterns were studied for three consecutive generations by following the strict self-pollination of mutant lines. Segregation analysis was performed in T_1_ generation for T-DNA-free mutant lines. We conducted the chi-square test for the confirmation of the Mendelian inheritance.

### 4.4. Phenotyping and Quantification of GA_3_

The data of major agronomic traits included: PH (plant height), PN (number of panicles), PL (length of panicle), SSR (rate of seed setting rate), GNPP (number of grain per panicle), FLW (width of flag leaf), FLL (length of flag leaf), GL (grains length), GW (1000-grain weight), GWD (grain width), and YPP (yield per plant). Endogenous GA levels were measured as previously described [75]. The measurement results were represented in μg/kg FW according to standard methods [101], with three replicates.

### 4.5. Microscopic Analysis and Application of GA_3_

Longitudinal sections of 0.5 cm taken from second internodes of mutant and WT mature plants and stained by using calcofluor and crystal violet and anatomical observations were done as described previously [102]. Slices were placed on slides in longitudinal sections and the results were analyzed by a Zeiss Axio Scope A1 Microscope. After 7 days of germination, the aqueous solution of GA_3_ with 10 μM concentration was applied by spraying on seedlings, with an equal quantity of pure water used as a control, and the plant height was recorded after 25 days.

### 4.6. Extraction, Digestion, and iTRAQ Labeling of Proteins

Proteins were extracted from 100 mg leaf samples of WT (VP-1643) and its CRISPR mutant GXU43_9, with three replicates, by grinding into liquid nitrogen, and immediately transferred to pre-cooled acetone (−20 °C), having 65 mM dithiothreitol (DTT), and 10% (*v*/*v*) TCA (trichloroacetic acid) was added and mixed thoroughly, precipitated (2 h at −20 °C), and centrifuged (16,000× *g* for 30 min at 4 °C). Supernatant was carefully removed, and the pellet was washed thrice with 20 mL pre-cooled acetone. It was centrifuged (20,000× *g* at 4 °C) and kept for half an hour at −20 °C. The precipitate was vacuum-dried soon after collection and the pellets obtained were fused with SDT containing 4% SDS, 100 mM Tris-HCl, 100 mM DTT, pH 8.0, boiled for 5 min, and then sonicated. The resultant product was centrifuged and filtered via a 0.22 μm Millipore filter. The concentration of proteins in the lysate was measured by using a bicinchoninic acid (BCA) assay kit (Beyo time Institute of Biotechnology, Shanghai, China). The extracted proteins were digested by following the FASP (filter-aided sample preparation) procedure [103]. iTRAQ labeling was conducted by using iTRAQ Reagents 8PLEXKit (Applied Biosystems, Foster City, CA, USA), according to the directions of the manufacturers. Peptides were labeled with iTRAQ tags as (GXU43_9)-114 and (WT1643)-115 and were combined and vacuum-dried at room temperature.

### 4.7. High-pH Reversed-phased Chromatography Separation

The fractionation of peptides was accomplished in a 1100 Series HPLC (high-performance liquid chromatography) System with a well-equipped Gemini-NX (Phenomenex, Torrance, CA, USA 00F-4453-E0) column (3 μm, 110 Å, 4.6 × 150 mm). The flow rate of 0.8 mL/min was maintained for the peptide’s elution. The composition of Buffer A was 10 mM mmonium acetate, and buffer B was 10 mM Ammonium acetate with a 90% *v*/*v* concentration of CAN and pH of 10, respectively. The gradient separation was used as follows: for 40 min, 100% buffer A, 3 min with 0–5% buffer B, 30 min with 5–35% buffer B, 10 min with 35–70% buffer B, 10 min with 70–75% buffer B, 7 min with 75–100% buffer B, 15 min with 100% buffer B, and finally, 15 min with 100% buffer A, and the absorbance was assessed at 214 nm. For every sample, 20 fractions were taken and merged to get ten fractions. After the vacuum centrifugation, fractions were reconstituted by trifluoroacetic 40 μL at a concentration of 0.1% *v*/*v*. The samples were stored at −80 °C until LC-MS/MS analysis.

### 4.8. LC-MS/MS Analysis

To analyze the peptides, an easy-nLC 1000 HPLC system attached to an Orbitrap Elite mass spectrometer (Thermo Fisher Scientific, San Jose, CA, USA) was used. The samples were loaded on the Thermo Scientific EASY column with an autosampler at 150 nL/min. Peptides separation was carried out by using a C18 trap column (inner diameter 100 μm × 2 cm) and a C18 analytical column (inner diameter 75 μm × 25 cm). After 120 min, segmented gradient ran with Buffer A (0.1% formic acid in water) to 35% Buffer B (0.1% formic acid in 100% ACN) for 100 min, followed by 35–90% Buffer B for 4 min, and finally, 90% Buffer B for 6 min. The MS was conducted at a high-resolution mode (60K), with MS scans ranging between 300 and 2000 m/z, and 20 signals were acquired from the MS spectra according to the abundance for MS/MS analysis. DDA (data-dependent acquisition) and HCD (higher-energy collisional dissociation) were exploited with a medium resolution (15K) in MS/MS. 50 ms (10 × 10^−6^) was the uppermost ion injection time, which was utilized for the survey scanned at 150 ms (5 × 10^4^) for the MS/MS scans respectively, while the duration of the dynamic exclusion was 30 s.

### 4.9. Data Analysis

We used Statistical Software Program SPSS 16.0 to analyze the data related to agronomic traits. Proteome Discoverer 2.1 was utilized for proteomics analysis against the Rice database (Oryza sativa subsp. Indica) on 17 September 2018, with 40,869 entries, by using the default parameters. Peptides with a global false discovery rate (FDR) <1% were used for further protein annotation. The DEPs were functionally annotated by the GO database (http://www.geneontology.org/) and Blast2go software (http://www.blast2go.com/b2ghome) and proteins were grouped according to their participation in the BP, CC, and MF. The DAPs (differentially accumulated proteins) were further assigned to the KEGG (http://www.genome.jp/kegg/pathway) database. Fisher’s Exact Test was used to identify the enriched GO terms and cluster analysis was performed by using Cluster 3.0 software. The pathways with FDR-corrected *p*-values ≤ 0.05 were regarded as significant. The PPI was evaluated by the STRING (http://string-db.org/) database and hub-proteins were assessed by Cytoscape (version 3.7.2).

### 4.10. Target Gene Expression Analysis and Proteomic Data Validation

We took 30-day-old rice seedlings and the panicle tissues for RNA extraction. RT-qPCR, performed on Real-Time LC480 (Roche Applied Science, Penzberg, Germany), polymerase chain reaction (PCR), was carried out by 10 μL volume with 0.08 μM primers, a 0.3 μL of reversed-transcribed product, and 5 μL of ChamQTM Universal SYBR qPCR Master Mix (Vazyme Biotech Co.,Ltd, Nanjing, China). The Rice *Actin* gene used as an internal control to normalize the reaction and the primers used are listed in Supplementary file 1, Table S1, and the comparative expression was evaluated by the 2^−ΔΔ^CT method, as established earlier [104].

## 5. Conclusions

In conclusion, we have explored that the guided mutations of *OsGA20ox2* through the CRISPR/Cas9 system is an effective strategy to develop semi-dwarf rice plants for sustainable production. Combining CRISPR/Cas9 and comprehensive proteomic analysis revealed new clues that will facilitate the understanding of the complex cellular and molecular events for important traits. In this study, fundamental resources are provided for identifying phytohormones, and some novel candidate proteins and metabolic pathways were found that could be involved in rice semi-dwarfism. The proteins crucial for various steps of GA and chlorophyll synthesis pathways were significantly repressed in semi-dwarf mutant lines. The results of this work revealed that the CRISPR/Cas9 technology is a very effective tool for the targeted gene editing, and these findings will contribute to an increased understanding of rice semi-dwarfism, and the generated mutant lines can be useful source material for future rice breeding programs.

## Figures and Tables

**Table 1:** Plant height (PH) and gibberellin (GA) content (μg/kg FW) in T_1_, T_2_, and T_3_ mutant lines.

Gen	Lines	GA_1_	GA_4_	PH
	WT	1.66 ± 0.14	1.54 ± 0.16	161.31 ± 3.9
	GXU43_2	1.19 ± 0.09 **	1.12 ± 0.12 **	128.25 ± 4.2 **
T_1_	GXU43_4	1.10 ± 0.10 **	1.05 ± 0.11 **	118.25 ± 2.9 **
	GXU43_9	0.95 ± 0.08 **	0.84 ± 0.10 **	114.77 ± 3.1 **
	GXU43_19	1.20 ± 0.12 **	1.24 ± 0.17 **	122.24 ± 3.1 **
	WT	1.59 ± 0.18	1.65 ± 0.15	164.45 ± 3.7
	GXU43_2	1.21 ± 0.11 **	1.24 ± 0.10 **	125.90 ± 4.3 **
T_2_	GXU43_4	1.20 ± 0.09 **	1.15 ± 0.12 **	117.53 ± 2.8 **
	GXU43_9	0.91 ± 0.11 **	0.81 ± 0.12 **	115.55 ± 3.5 **
	GXU43_19	1.14 ± 0.13 **	1.30 ± 0.13 **	123.35 ± 3.3 **
	WT	1.60 ± 0.15	1.63 ± 0.17	163.31 ± 3.9
	GXU43_2	1.14 ± 0.10 **	1.29 ± 0.11 **	126.25 ± 4.2 **
T_3_	GXU43_4	1.20 ± 0.12 **	1.35 ± 0.13 **	116.25 ± 2.9 **
	GXU43_9	0.88 ± 0.13 **	0.87 ± 0.12 **	113.98 ± 3.1 **
	GXU43_19	1.21 ± 0.15 **	1.22 ± 0.14 **	121.24 ± 3.1 **

Gen: generations, FW: fresh weight, PH: plant height, GA: gibberellins. Mean data for three independent replicates. ** represent a significant difference at *p* ≤ 0.01.

**Table 2:** Performance of major agronomic traits in WT and mutant lines.

Gen	Line	PN	PL (cm)	FLL (cm)	FLW (cm)	GNPP	SSR (%)	GW (g)	YPP (g)	GL mm	GWD mm
	WT	9.91 ± 0.8	27.16 ± 1.4	49.97 ± 3.39	1.75 ± 0.1	145 ± 12	87.12 ± 5.9	29.34 ± 1.3	31.53 ± 2.1	8.52 ± 0.4	2.41 ± 0.2
	GXU43_2	9.17 ± 0.3 ^ns^	26.41 ± 1.3 ^ns^	38.63 ± 2.40 **	1.61 ± 0.2 ^ns^	148 ± 10 ^ns^	92.10 ± 4.1 ^ns^	29.63 ± 1.8 ^ns^	31.85 ± 1.3 ^ns^	8.19 ± 0.3 ^ns^	2.49 ± 0.3 ^ns^
T_1_	GXU43_4	10.28 ± 0.5 ^ns^	26.15 ± 2.5 ^ns^	39.13 ± 2.13 **	1.71 ± 0.3^ns^	147 ± 11 ^ns^	91.33 ± 5.1 ^ns^	30.27 ± 1.1 ^ns^	30.41 ± 1.2 ^ns^	8.61 ± 0.4 ^ns^	2.35 ± 0.4 ^ns^
	GXU43_9	10.29 ± 0.6 ^ns^	24.98 ± 1.9 ^ns^	37.43 ± 2.15 **	1.64 ± 0.1 ^ns^	149 ± 09 ^ns^	90.44 ± 5.3 ^ns^	30.29 ± 2.2 ^ns^	32.53 ± 1.4 ^ns^	9.10 ± 0.4 ^ns^	2.40 ± 0.4 ^ns^
	GXU43_19	10.75 ± 0.4 ^ns^	25.30 ± 1.3 ^ns^	40.33 ± 1.85 **	1.55 ± 0.3 ^ns^	146 ± 12 ^ns^	93.03 ± 5.7 ^ns^	30.43 ± 1.6 ^ns^	30.95 ± 1.5 ^ns^	8.98 ± 0.5 ^ns^	2.45 ± 0.3 ^ns^
	WT	10.81 ± 0.9	28.12 ± 1.8	48.59 ± 3.88	1.90 ± 0.2	147 ± 13	88.62 ± 6.5	29.72 ± 1.5	31.21 ± 1.8	8.95 ± 0.5	2.39 ± 0.3
	GXU43_2	10.63 ± 0.5 ^ns^	26.63 ± 1.9 ^ns^	39.84 ± 2.69 **	1.75 ± 0.3 ^ns^	146 ± 11 ^ns^	93.30 ± 5.4 ^ns^	29.96 ± 1.7 ^ns^	32.44 ± 1.9 ^ns^	8.27 ± 0.3 ^ns^	2.33 ± 0.2 ^ns^
T_2_	GXU43_4	9.71 ± 0.7 ^ns^	25.55 ± 2.1 ^ns^	38.23 ± 2.52 **	1.65 ± 0.5 ^ns^	149 ± 12 ^ns^	90.95 ± 6.2 ^ns^	30.42 ± 1.3 ^ns^	31.77 ± 1.5 ^ns^	8.06 ± 0.5 ^ns^	2.29 ± 0.3 ^ns^
	GXU43_9	10.29 ± 0.8 ^ns^	26.88 ± 1.3 ^ns^	36.70 ± 2.58 **	1.55 ± 0.2 ^ns^	150 ± 12 ^ns^	92.51 ± 6.9 ^ns^	30.88 ± 2.1 ^ns^	32.56 ± 1.7 ^ns^	8.87 ± 0.7 ^ns^	2.48 ± 0.3 ^ns^
	GXU43_19	10.85 ± 0.6 ^ns^	24.99 ± 1.7 ^ns^	39.73 ± 2.85 **	1.71 ± 0.3 ^ns^	148 ± 10 ^ns^	91.43 ± 6.6 ^ns^	29.54 ± 1.9 ^ns^	31.73 ± 1.6 ^ns^	8.61 ± 0.6 ^ns^	2.38 ± 0.4 ^ns^
	WT	10.50 ± 0.8	27.96 ± 1.6	50.97 ± 3.39	1.85 ± 0.1	146 ± 12	86.85 ± 5.9	30.14 ± 1.3	31.96 ± 2.6	8.34 ± 0.7	2.27 ± 0.2
	GXU43_2	10.90 ± 0.3 ^ns^	27.41 ± 1.3 ^ns^	40.63 ± 2.40 **	1.67 ± 0.2 ^ns^	149 ± 10 ^ns^	90.10 ± 6.1 ^ns^	30.33 ± 1.8 ^ns^	31.15 ± 1.3 ^ns^	8.45 ± 0.5 ^ns^	2.31 ± 0.3 ^ns^
T_3_	GXU43_4	10.77 ± 0.5 ^ns^	24.86 ± 1.5 ^ns^	41.33 ± 2.13 **	1.76 ± 0.3 ^ns^	147 ± 11 ^ns^	92.33 ± 5.8 ^ns^	29.92 ± 1.7 ^ns^	30.49 ± 1.2 ^ns^	8.62 ± 0.4 ^ns^	2.42 ± 0.4 ^ns^
	GXU43_9	10.45 ± 0.6 ^ns^	24.18 ± 1.9 ^ns^	38.40 ± 2.15 **	1.62 ± 0.1 ^ns^	150 ± 09 ^ns^	93.44 ± 5.3 ^ns^	30.50 ± 2.2 ^ns^	31.85 ± 1.4 ^ns^	8.50 ± 0.6 ^ns^	2.41 ± 0.4 ^ns^
	GXU43_19	10.99 ± 0.4 ^ns^	25.30 ± 1.7 ^ns^	39.33 ± 2.50 **	1.59 ± 0.3 ^ns^	149 ± 11 ^ns^	89.03 ± 5.7 ^ns^	29.93 ± 1.6 ^ns^	31.29 ± 1.5 ^ns^	8.68 ± 0.5 ^ns^	2.36 ± 0.3 ^ns^

Gen: Generation; WT: wild-type; PL: panicle length, PN: panicle numbers; FLW: flag leaf width, FLL: flag leaf length, GNPP: grain number per panicle; SSR: seed setting rate; GW: 1000-grain weight; YPP: yield per plant; GWD: grain width; GL: grain length. Mean data of three replicates. ** represent the significant difference and ^ns^ represents the non-significant difference at *p* < 0.01.

**Table 3:** Differentially expressed proteins related to GA and plant growth.

Protein ID	Locus/Gene Name	Annotation	Regulate
Q6AWY7	*Os06g0204800/GRF2*	Growth-regulating factor 2	Down
Q6AWY2	*Os12g0484900/GRF7*	Growth-regulating factor 7	Down
Q9FRG8	*Os03g0674700/GRF9*	Growth-regulating factor 9	Down
Q6EPP9	*Os02g0678800/GRF10*	Growth-regulating factor 10	Down
Q6AWX8	*Os07g0467500/GRF11*	Growth-regulating factor 11	Down
Q8RZ73	*B1065G12.22*	Gibberellin response modulator-like proteins	Down
Q9AS97	*Os01g0646300*	Gibberellin response modulator-like	Down
Q69VG1	*Os07g0545800/CIGR1*	Chitin-inducible gibberellin-responsive protein 1	Down
Q339D4	*LOC_Os10g22430*	Chitin-inducible gibberellin-responsive protein 2	Down
Q8LNJ6	*OSJNBb0028C01.33*	Putative gibberellin oxidase	Down
Q0JH50	*Os01g0883800/GA20ox2*	Gibberellin 20 oxidase 2	Down
Q5MQ85	*CPS2*	Ent-copalyl diphosphate synthase 2	Down
P0C511	*RBCS*	Ribulose bisphosphate carboxylase large chain	Down
A2YQT7	*GAPC*	Glyceraldehyde-3-phosphate dehydrogenase, cytosolic	Down
Q6ZG90	*Os02g0131300*	ATP synthase	Down
J7EYN3	*atpB*	ATP synthase subunit beta, chloroplastic	Down
A2XEX2	*OsI_10887*	Fructose-1,6-bisphosphatase, chloroplastic	Down
C6L7X5	*Snorkel2*	Ethylene response factor	Down
Q53JF7	*Os11g0167800/ASR5*	Abscisic stress-ripening protein	Up
Q6I5C3	*Os05g0213500/PYL5*	Abscisic acid receptor	Up

## Supplementary Materials

The following are available online at https://www.mdpi.com/1422-0067/21/17/6170/s1: Table S1: All the primers used in this study; Table S2: Type of mutations obtained in T_0_ generation by two constructs of CRISPR/Cas9; Table S3: Mutations detection on the potential off-targets; Table S4: Segregation of mutations induced by CRISPR/Cas9 in target genes; Table S5: Positions and efficiency score of both the targets; Table S6: List of primers utilized for analyzing off-targets. Figure S1: PCR amplification of CRISPR/Cas9 T-DNA integration; Figure S2: Analysis of the proteome of wild type (WT) and CRISPR/Cas9 mutants of rice.; Figure S3: Structure of pYLCRISPR/Cas9Pubi-*H* binary vector with fragment containing a modified ccdB flanked by two BsaI sites; Figure S4: Embedded view of plasmids; Figure S5: Schematic diagram of Vector map and sgRNA target sites in *OsGA20ox2*; Figure S6: Schematic representation of secondary structures; Figure S7: Illustration of overlapping PCR for generation of expression cassette; Figure S8: Detection and amplification of CRISPR/Cas9 T-DNA integration and the *OsGA20ox2* target sequence assembly in vector.
