# Supplementary material for: CRISPR/Cas9 Directed Mutagenesis of OsGA20ox2 in High Yielding Basmati Rice (Oryza sativa L.) Line and Comparative Proteome Profiling of Unveiled Changes Triggered by Mutations
Source: Int J Mol Sci. 2020 Aug 26;21(17):6170. doi: 10.3390/ijms21176170 (PMC7504442; doi:10.3390/ijms21176170)
Supplement: Supplementary file 1 [file ijms-21-06170-s001.zip › supplementary file 1.pdf]

International Journal of *Molecular Sciences*

MDPI

*Supplementary*

# CRISPR/Cas9 Directed Mutagenesis of *OsGA20ox2* in High Yielding Basmati Rice (*Oryza sativa* L.) Line and Comparative Proteome Profiling of Unveiled Changes Triggered by Mutations

**Gul Nawaz [1], Babar Usman [1], Neng Zhao [1], Yue Han [1], Zhihua Li [1], Xin Wang [1], Yaoguang Liu [2,*] and Rongbai Li [1,*]**

[1] College of Agriculture, State Key Laboratory for Conservation and Utilization of Subtropical Agro-Bioresources, Guangxi University, Nanning 530004, China; gulnawazmalik@yahoo.com (G.N.); babarusman119@gmail.com (B.U.); nengzhao_gxu@163.com (N.Z.); hanyue0624@126.com (Y.H.); lizhihua-88@163.com (Z.L.); xinwang0112@126.com (X.W.)

[2] State Key Laboratory for Conservation and Utilization of Subtropical Agricultural Bioresources, South China Agricultural University, Guangzhou 510642, China

* Correspondence: ygliu@scau.edu.cn (Y.L.); lirongbai@126.com (R.L.); Tel.: +86-20-8528-1908 (Y.L.); +86-136-0009-4135 (R.L.)

**Abstract:** In rice, semi-dwarfism is among the most required characteristics, as it facilitates better yields and offers lodging resistance. Here, semi-dwarf rice lines lacking any residual transgene-DNA and off-target effects were generated through CRISPR/Cas9-guided mutagenesis of the *OsGA20ox2* gene in a high yielding Basmati rice line, and the isobaric tags for relative and absolute quantification (iTRAQ) strategy was utilized to elucidate the proteomic changes in mutants. The results indicated the reduced gibberellins ($GA_1$ and $GA_4$) levels, plant height (28.72%), and flag leaf length, while all the other traits remained unchanged. The *OsGA20ox2* expression was highly suppressed, and the mutants exhibited decreased cell length, width, and restored their plant height by exogenous $GA_3$ treatment. Comparative proteomics of the wild-type and homozygous mutant line (GXU43_9) showed an altered level of 588 proteins, 273 upregulated and 315 downregulated, respectively. The identified differentially expressed proteins (DEPs) were mainly enriched in the carbon metabolism and fixation, glycolysis/gluconeogenesis, photosynthesis, and oxidative phosphorylation pathways. The proteins (Q6AWY7, Q6AWY2, Q9FRG8, Q6EPP9, Q6AWX8) associated with growth-regulating factors (*GRF2*, *GRF7*, *GRF9*, *GRF10*, and *GRF11*) and GA (Q8RZ73, Q9AS97, Q69VG1, Q8LNJ6, Q0JH50, and Q5MQ85) were downregulated, while the abscisic stress-ripening protein 5 (*ASR5*) and abscisic acid receptor (*PYL5*) were upregulated in mutant lines. We integrated CRISPR/Cas9 with proteomic screening as the most reliable strategy for rapid assessment of the CRISPR experiments outcomes.

**Keywords:** rice; gibberellins; plant height; CRISPR/Cas9; *OsGA20ox2*; proteomic analysis

**Table S1.** All the primers used in this study.

| Primer name | Primer sequence (5'-3') |
|---|---|
| Target 1 | AGATCCCGGAGCCATTCGTGTGG |
| Target 2 | GGCCCCGACTTCGCGCCAATGGG |
| *SD1T1 F:* | AATCTCATGGTGGCCGAGC |
| *SD1T1 R:* | CGGGAGGCGGAAGAAGTC |
| *SD1T2 F:* | CACGCACGGGTTCTTCC |
| *SD1T2 R:* | TCCATGATCGTCAGCGACAG |
| U-F | CTCCGTTTTACCTGTGGAATCG |
| gR-R | CGGAGGAAAATTCCATCCAC |
| U6aTSD1-1-R | CACGAATGGCTCCGGGATCTCggcagccaagccagca |
| gRTSD1-1-F | AGATCCCGGAGCCATTCGTGgttttagagctagaaat |
| U6bT1SD1-2-R | ATTGGCGCGAAGTCGGGGCCaacacaagcggcagc |
| gRT1SD1-2-F | GCCCCGACTTCGCGCCAATgttttagagctagaaat |
| Pps-GGL | TTCAGAGGTCTCTCTCGACTAGTATGGAATCGGCAGCAAAGG |
| Pgs-GG2 | AGCGTGGGTCTCGTCAGGGTCCATCCACTCCAAGCTC |
| Pps-GG2 | TTCAGAGGTCTCTCTGACACTGGAATCGGCAGCAAAGG |
| Pgs-GGR | AGCGTGGGTCTCGACCGACGCGTATCCATCCACTCCAAGCTC |
| *HPT F:* | GTGCTTGACATTGGGGAGTT |
| *HPTR:* | ATTTGTGTACGCCCGACAGT |
| *Actin-F* | GAGTATGATGAGTCGGGTCCAG |
| *Actin-R* | ACACCAACAATCCCAAACAGAG |
| *Cas9-F* | CTGACGCTAACCTCGACAAG |
| *Cas9-R* | CCGATCTAGTAACATAGATGACACC |
| *SD1-F* | GCCAATGGGGAGGGTGTAC |
| *SD1-R* | CTGTAGTAGCCTCGCTCCAC |
| *SP-L1* | GCGGTGTCATCTATGTTACTAG |
| *SP-R* | GCCTATACCAAGTTATTGCA |
| *CPS2 F* | CATCTCCAAGGTTTTGTTCGAG |
| *CPS2 R* | TTTTCGCAGTCATCACTTTACG |
| *GRF2 F* | GACCGATGCTTTCACTCACC |
| *GRF2 R* | CTGAGGATTGCAGCAACCAA |
| *RBCS-F* | TCATTGGCTTTGACAACGTTAG |
| *RBCS-R* | AAAGAAAGAACATGCACGAAGG |
| *CIGR1 F* | TACCTCTCCTCCCGATGACA |
| *CIGR1 R* | AATCAACGTCGCCTTCATCG |
| *ASR5-F* | CTGTTTTTCACAAGAGTCTCCG |
| *ASR5-R* | CACCACTTATTTGGACACACAG |
| *atpB-F* | GCCCGTCGGAAAGTTCAATC |
| *atpB-R* | TAGCGCAACCCCAAATCAAC |
| *GRF7 F* | ATCTAGCCATGCCGCAAAAG |
| *GRF7 R* | TCTATTGGGCAGCAGTCACA |
| *GRF10 F* | GGTGCTCATCTACCGCTACT |
| *GRF10 R* | AAACGGCGTCCACATTATCG |
| *Snorkel2-F* | AGAACGATAACAATGGCGCC |
| *Snorkel2-R* | GAGTGTGGTGTTTTCGCAGT |
| *PYL5 F* | CTGTCTCCCCACTCCTTCAG |
| *PYL5 R* | GGCCTCGACAAAGTAGCATG |

Note: ACTAGT and ACGCGT: *SpeI* and *MluI* restriction enzyme cutting sites.

**Table S2.** Type of mutations obtained in $T_0$ generation by two constructs of CRISPR/Cas9.

| Targets/sgRNA | Event ID | Zygosity | Type of mutations | Copy# | T-DNA |
|---|---|---|---|---|---|
| Target 1 | GXU43_2 | Homozygous | 1i/1i | 1 | + |
| | GXU43_4 | Homozygous | 27d/27d | 1 | + |
| | GXU43_6 | Biallelic | 6d/1i | 4 | + |
| | GXU43_7 | Heterozygous | 6d/WT | 2 | + |
| | GXU43_8 | Heterozygous | WT/1i | 2 | + |
| | GXU43_9 | Homozygous | 172d/172d | 3 | + |
| | GXU43_16 | Chimeric | 19d/1i/WT | 1 | + |
| | GXU43_19 | Homozygous | 4d/4d | 3 | + |
| | GXU43_24 | Biallelic | 4d/8d | 3 | + |
| | GXU43_30 | Biallelic | 22d/1i15d | 2 | + |
| Target 2 | GXU43_1 | Biallelic | d1/d5 | 1 | + |
| | GXU43_2 | Homozygous | 3d/3d | 1 | + |
| | GXU43_4 | Homozygous | 1d/1d | 2 | + |
| | GXU43_8 | Biallelic | 4d/2d | 2 | + |
| | GXU43_9 | Homozygous | 12d/12d | 1 | + |
| | GXU43_10 | Heterozygous | 4d/WT | 1 | + |
| | GXU43_15 | Biallelic | 4d/1i | 3 | + |
| | GXU43_19 | Homozygous | 1i/1i | 3 | + |
| | GXU43_20 | Biallelic | 3d/1d | 1 | + |
| | GXU43_23 | Biallelic | 5d/1d | 1 | + |
| | GXU43_25 | Chimeric | WT/5d/13d | 2 | + |
| | GXU43_29 | Biallelic | 1d/2d | 2 | + |

d: deletion, i: insertion and WT: wild type. The numbers in front of the letters indicate the number of nucleotides affected. Corresponding mutations in two alleles are distinguished by '/'.

**Table S3.** Mutations detection on the potential off-targets.

| Target | Name of putative off-target site | Genomic location | Sequence of the putative off-target site | Gene Locus | No. of mismatching bases | No. of plants sequenced | No. of plants with mutations |
|---|---|---|---|---|---|---|---|
| *SD1T1* | OT1 | Chr5: 13412868 | AGATCC**A**GGAG**A**CA**A**T**T**GTG **AGG** | LOC_Os05g23450 | 4 | 30 | 0 |
| | OT2 | Chr12: 5293287 | AGA**ATA**CG**A**AGCCATTC**T**TG **TGG** | LOC_Os12g09990 | 5 | 30 | 0 |
| | OT3 | Chr11: 17594551 | AG**G**TC**G**CGG**T**G**G**CAT**C**CGTG **TGG** | LOC_Os11g30280 | 5 | 30 | 0 |
| | OT4 | Chr5: 20691896 | AGATCCCGG**C**GCC**G**TTCGT**C** **TGG** | LOC_Os05g34854 | 3 | 30 | 0 |
| | OT5 | Chr4: 2035446 | AGATCCCGGAG**T**C**G**TTC**T**CG **AGG** | LOC_Os04g04330 | 3 | 30 | 0 |
| SD1T2 | OT6 | Chr8: 5222727 | **C**GC**AT**CGACT**A**CGCGCCAAT **GGG** | LOC_Os08g08970 | 4 | 30 | 0 |
| | OT7 | Chr2: 3673463 | **AC**CC**A**CG**G**CTTCGCGCC**G**AT **GGG** | LOC_Os02g07140 | 4 | 30 | 0 |
| | OT8 | Chr9: 4990938 | **A**GCC**A**CG**G**CTT**T**GCGC**A**AAT **GGG** | LOC_Os09g09320 | 5 | 30 | 0 |
| | OT9 | Chr3:4617541 | GGCCCCGACTTCGC**CG**C**C**AT **GCG** | LOC_Os03g08920 | 4 | 30 | 0 |
| | OT10 | Chr3: 8521554 | GGCCC**G**GACTT**G**GCGC**TG**AT **GGT** | LOC_Os03g15520 | 4 | 30 | 0 |

Note: The protospacer adjacent motif (PAM) (NGG) is shown in green background.

**Table S4.** Segregation of mutations induced by CRISPR/Cas9 in target genes.

| T0 | | | | | T1 | | | |
|---|---|---|---|---|---|---|---|---|
| **Targets/sgRNA** | **Mutants** | **Zygosity** | **Type of mutations** | **No. of plants tested** | **WT** | **Bi** | **Homo** | **Hetero** |
| Target 1 | GXU43_2 | Homozygous | 1i/1i | 40 | 0 | 0 | 40(1i/1i) | 0 |
| | GXU43_4 | Homozygous | 27d/27d | 40 | 0 | 0 | 40(27d/27d) | 0 |
| | GXU43_8 | Heterozygous | WT/1i | 40 | 10 | 0 | 12(1i) | 18(WT/1i) |
| | GXU43_9 | Homozygous | 172d/172d | 40 | 0 | 0 | 40(172d/172d) | 0 |
| | GXU43_19 | Homozygous | 4d/4d | 40 | 0 | 0 | 40(4d/4d) | 0 |
| Target 2 | GXU43_2 | Homozygous | 3d/3d | 40 | 0 | 0 | 40(3d/3d) | 0 |
| | GXU43_4 | Homozygous | 1d/1d | 40 | 0 | 0 | 40(22d/22d) | 0 |
| | GXU43_8 | Biallelic | 4d/2d | 40 | 0 | 19(4d/2d) | 11(4d), 10(2d) | 0 |
| | GXU43_9 | Homozygous | 12d/12d | 40 | 0 | 0 | 40(12d/12d) | 0 |
| | GXU43_19 | Homozygous | 1i/1i | 40 | 0 | 0 | 40(1i/1i) | 0 |

**Table S5.** Positions and efficiency score of both the targets.

| Target Name | Position | Strand | Off Target Score | GC (%) | Region | Pairing with SgRNA |
|---|---|---|---|---|---|---|
| Target1 | 128–147 | + | 0.145 | 60.0 | CDS | None |
| Target2 | 541–560 | + | 0.287 | 70.0 | CDS | None |

**Table S6.** List of primers utilized for analyzing off-targets.

| Primer name | Primer sequence (5'-3') |
|---|---|
| POT1 | F: CAAGGGCCCATGAGCATAAC<br>R: GTTGCCGGATAGAACAGCTG |
| POT2 | F: CAAGTCTCAAGTGCTGGCTG<br>R: AGAGCCCCTGGTGTATTTCC |
| POT3 | F: TGCTACCTTGCCGAGATGTA<br>R: TCATCCGGTGCAATCCTGTA |
| POT4 | F: GAAGGGAGGAGGAGGAGAAG<br>R: AGGGTGGAGAAGTAATCGGC |
| POT5 | F: GACCTCCTGCTCTTCGACAA<br>R: GTTGTTGTCGCTGAGCATGA |
| POT6 | F: CACCTGTGCGAGTAAATGGG<br>R: AGAGATTGGTGGCGTTGATCC |
| POT7 | F: GATGCGAGTACGAGTCAATC<br>R: CTACTGCTGCTGCTGCTGCG |
| POT8 | F: TGTTTGCAAAATACCGTTTCGA<br>R: GAAGATGTGTGCGCATGTCT |
| POT9 | F: TTCTCCGTCCTCATCAACCG<br>R: CTCCTCCGACTCTTCCTCCT |
| POT10 | F: TGTACTGCGAGGCTGATCAA<br>R: TCGATGTTCCACGGGTTAGG |

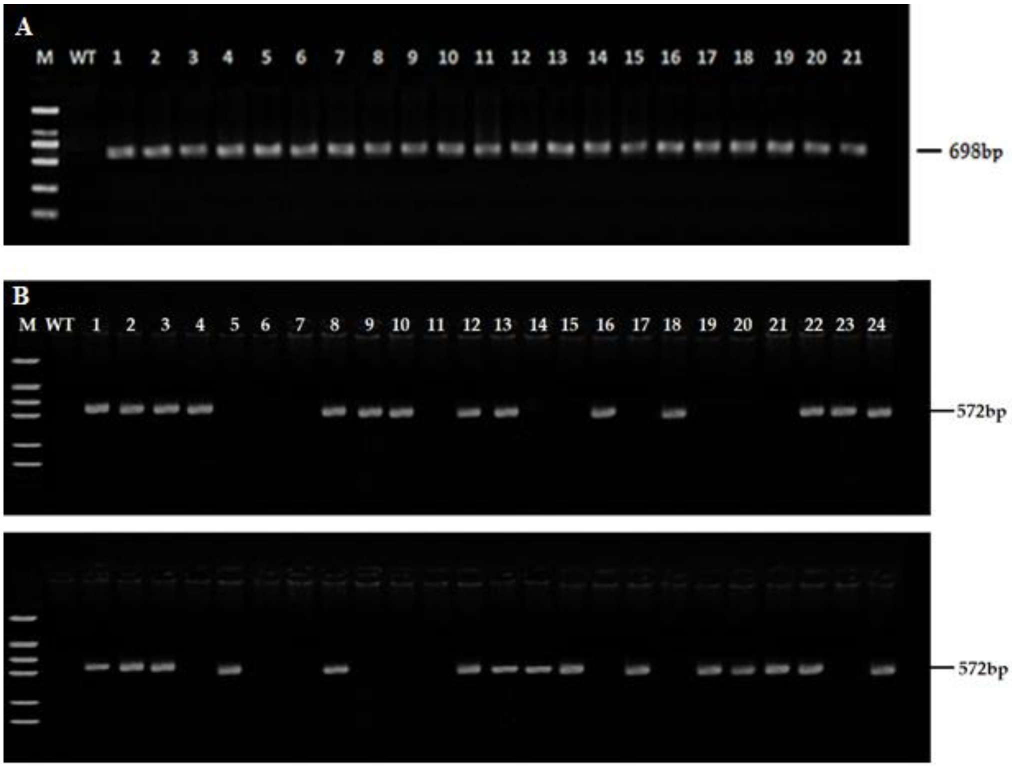


**Figure S1.** PCR amplification and detection of CRISPR/Cas9 T-DNA integration and the *OsGA20ox2* target sequence assembled in pYLCRISPR/Cas9Pubi-*H*. (**A**) Detection of T0 positive mutant lines; M: DL2000 DNA marker; WT: wild type; (**B**) Assessment of transgene free mutant lines; M: DL2000 DNA marker; WT: wild type.

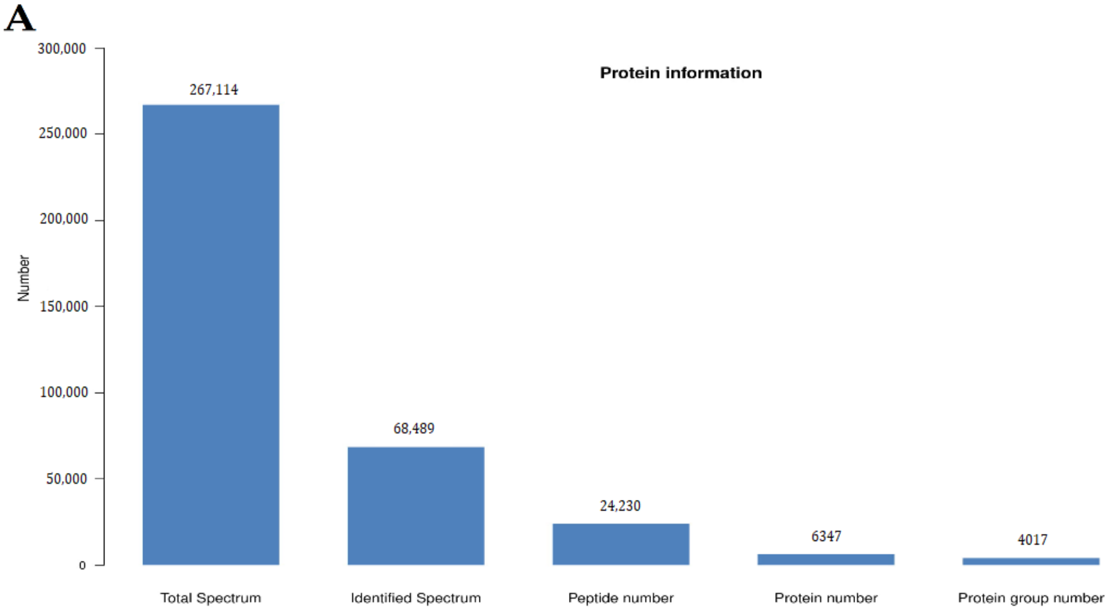


***Cont.*** Figure S2.

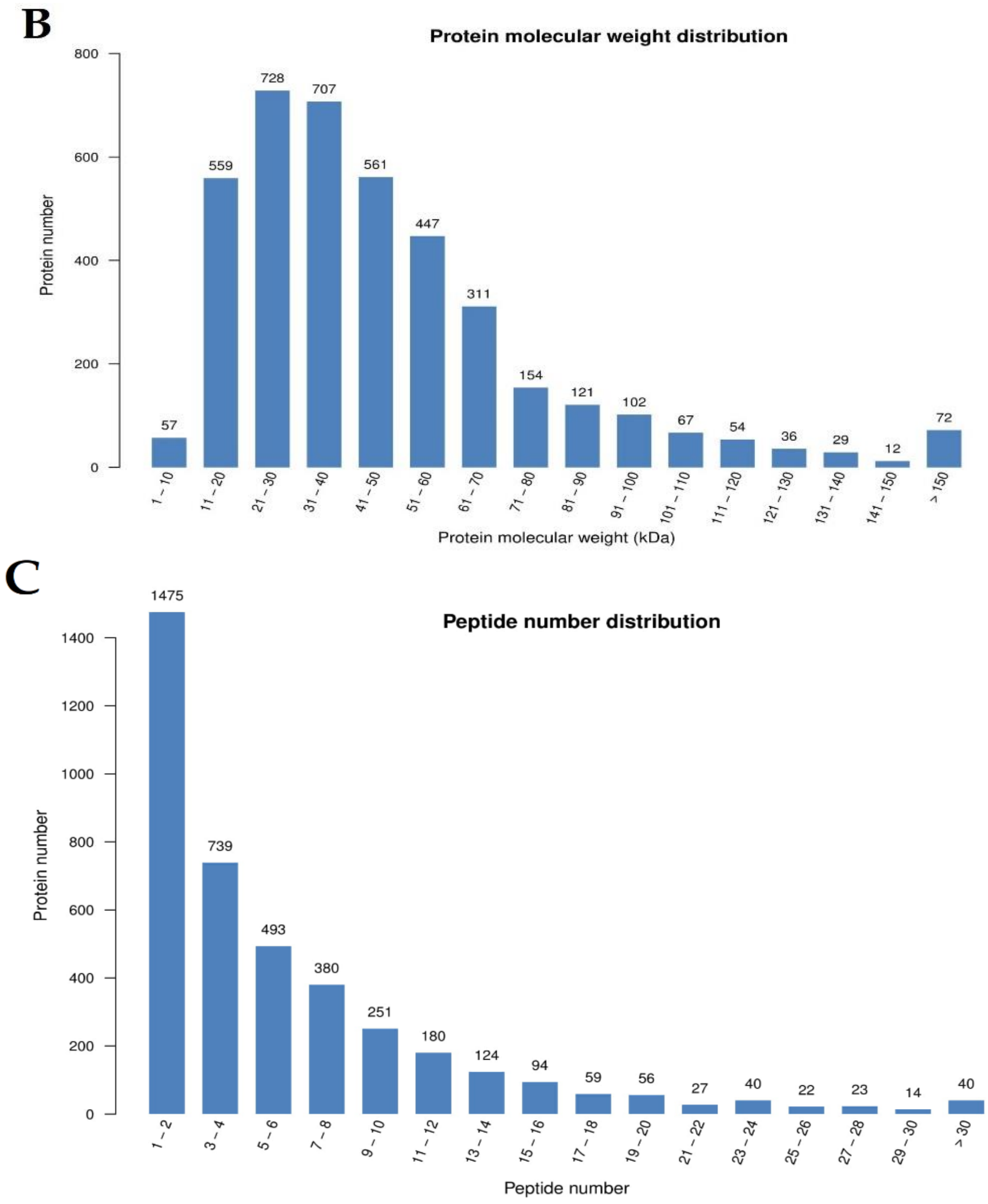


***Cont.*** Figure S2.

D

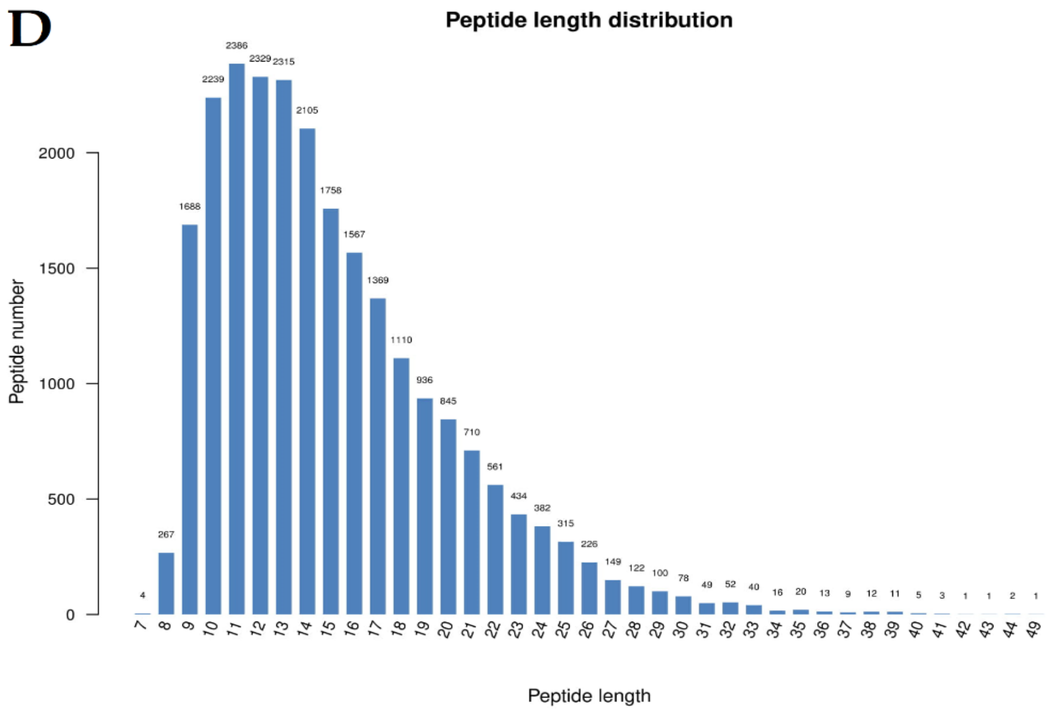


*Cont.* Figure S3.

E

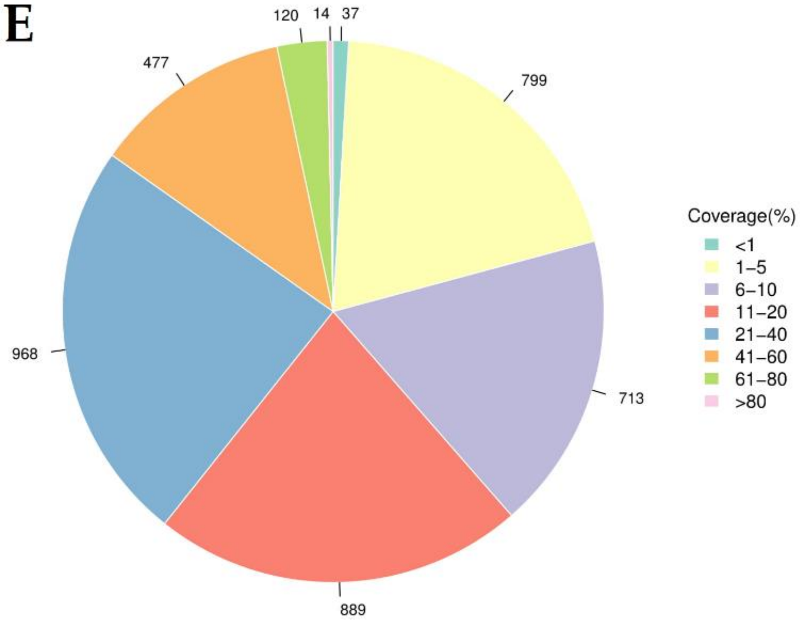


*Cont.* Figure S2.

F

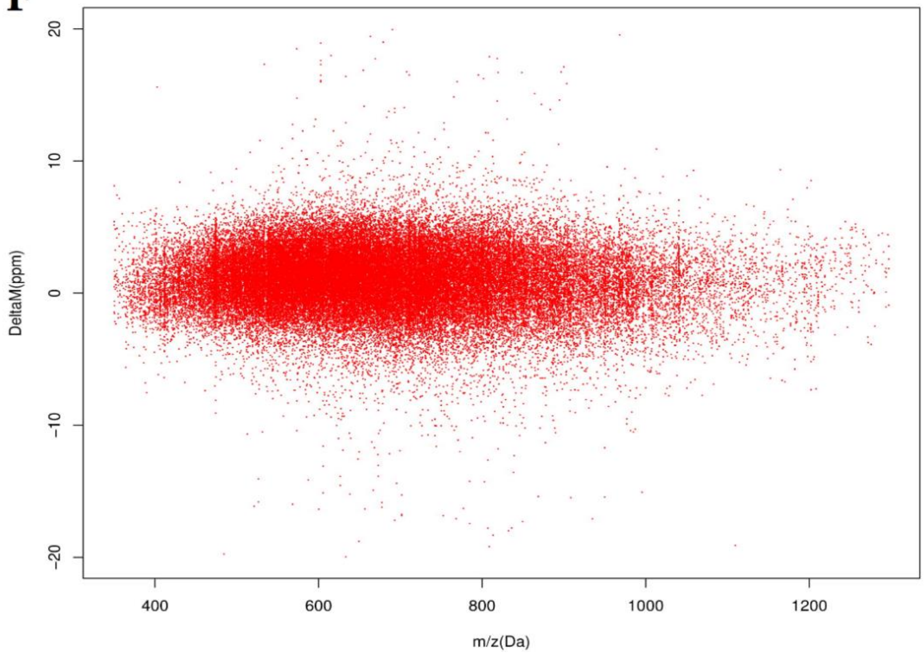


**Figure S2.** Analysis of the proteome of wild type (WT) and CRISPR/Cas9 mutants of rice. (**A)** Protein information histogram. The x-axis is the information of the identified protein. From left to right: total spectrum of MS/MS, the number of matched spectra the peptide number, the protein number and the protein group number of identified proteins were grouped based on their protein mass; (**B)** Protein molecular weight distribution histogram. The X-axis is the molecular weight of the identified protein. Y-axis is the number of identified proteins; (**C)** Peptide number distribution histogram. X-axis is the peptide number of identified proteins. Y-axis is the number of identified proteins; (**D)** Peptide length distribution. X-axis is the length of the identified peptide which represents the number of amino acid residues in the peptide. Y-axis is the number of peptides matched its length; (**E)** Protein coverage distribution pie chart. Each fan represents the proportion of the protein coverage in all identified proteins. The larger area in the fans, the greater proteins in the protein coverage. The numbers represent the number of the proteins in this area; (**F)** Matching error along peptide. X-axis is the mass-to-charge ratio, which represents the ratio of fragmented ions' mass with charge. Y-axis presents the matching error of fragmented ions' mass. For protein identification, a mass tolerance of 10 ppm was permitted for intact peptide masses.

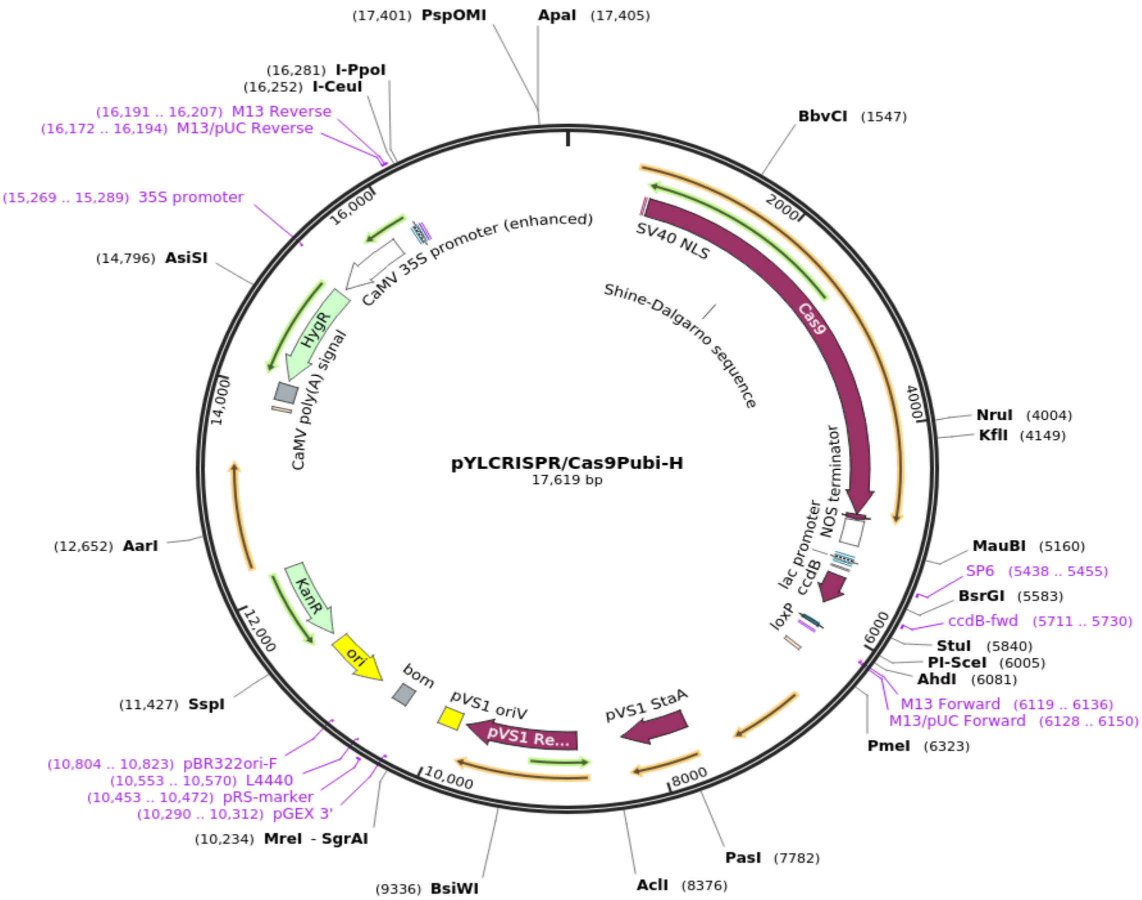


**Figure S3.** Structure of pYLCRISPR/Cas9Pubi-H binary vector with fragment containing a modified ccdB flanked by two *BsaI* sites.

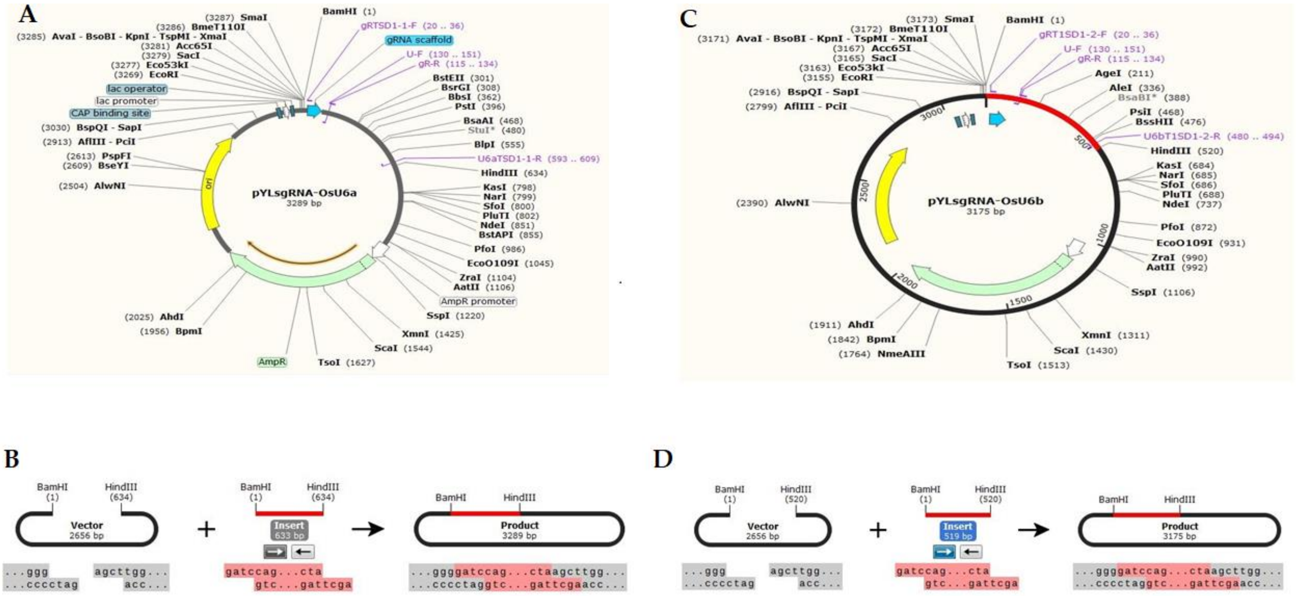


**Figure S4.** Embedded view of plasmids (**A**) pYLsgRNA-OsU6a (3289bp); (**B**) Cloning strategy of PYLsgRNA-OsU6a showing sticky ends created by cutting with two enzymes (BamH1 and HindIII), selected fragments replaced indicated in white and the remaining vector fragment is in black (**C**) pYLsgRNA-OsU6b (3175bp) (**D**) Cloning strategy of PYLsgRNA-OsU6b showing sticky ends created by cutting with two enzymes (BamH1 and HindIII), selected fragments replaced indicated in white and the remaining vector fragment is in black.

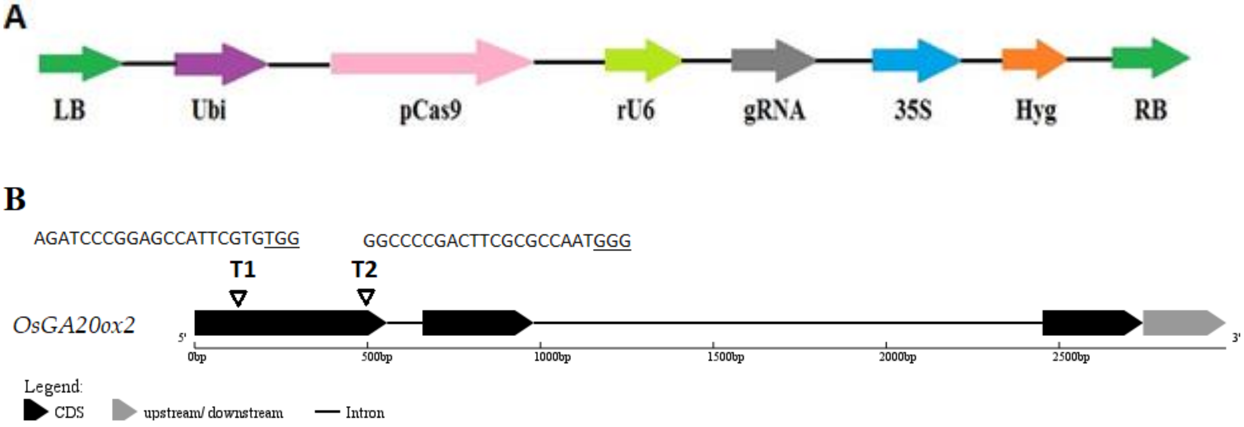


**Figure S5.** Schematic diagram of Vector map and sgRNA target sites in *OsGA20ox2*. (**A**) Vector map of Cas9/gRNA. LB: Vector left border; Ubi: ubiquitin promoter; pCas9, Cas9 protein; gRNA: Guided RNA; rU6: Rice U6 promoter; 35S: CaMV 35S promoter; Hyg: Hygromycin; RB, Vector right border; (**B**) Exons are indicated as black boxes. $T_1$ and $T_2$ represent Target 1 and Target 2, respectively. Target 1 was from 128–147 bp and Target 2 was 541–560bp in the first exon.

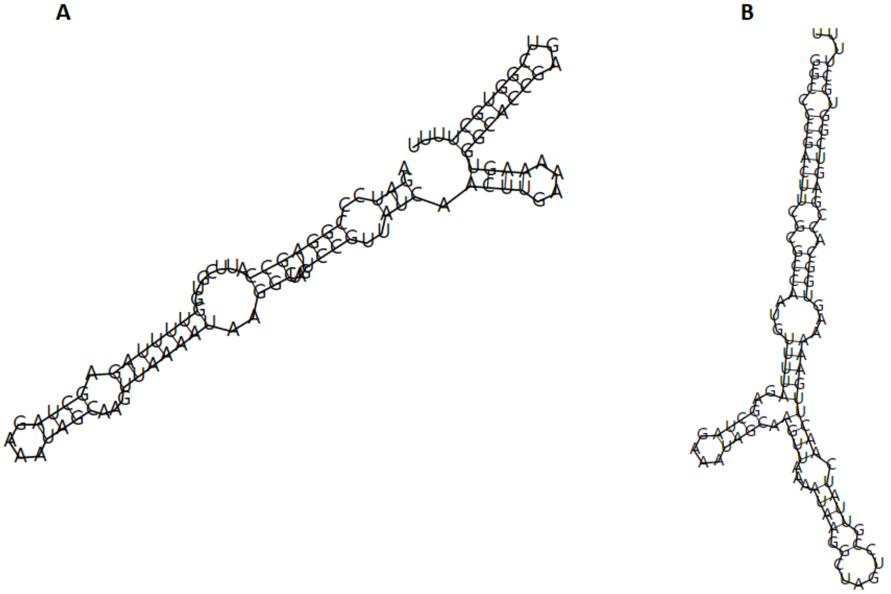


**Figure S6.** Schematic representation of secondary structures (**A)** sgRNA1 and (**B)** sgRNA2, used in for experiment. The secondary structure of both sgRNAs was developed by CRISPR P (http://crispr.hzau.edu.cn/cgi-bin/CRISPR/CRISPR).

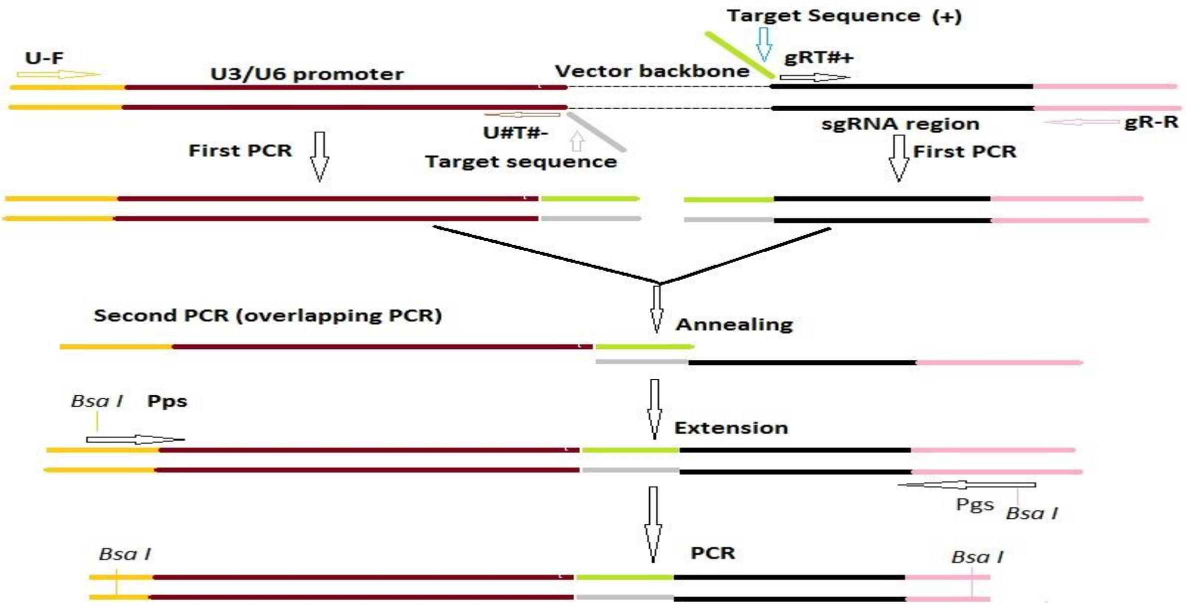


**Figure S7.** Illustration of overlapping PCR for generation of expression cassette. U-F/U#T#- and gRT#+/gR-R primers are used in separately in both reactions. U#, T#+, and T#- indicates a given promoter, forward and reverse strands of a target sequence, respectively.

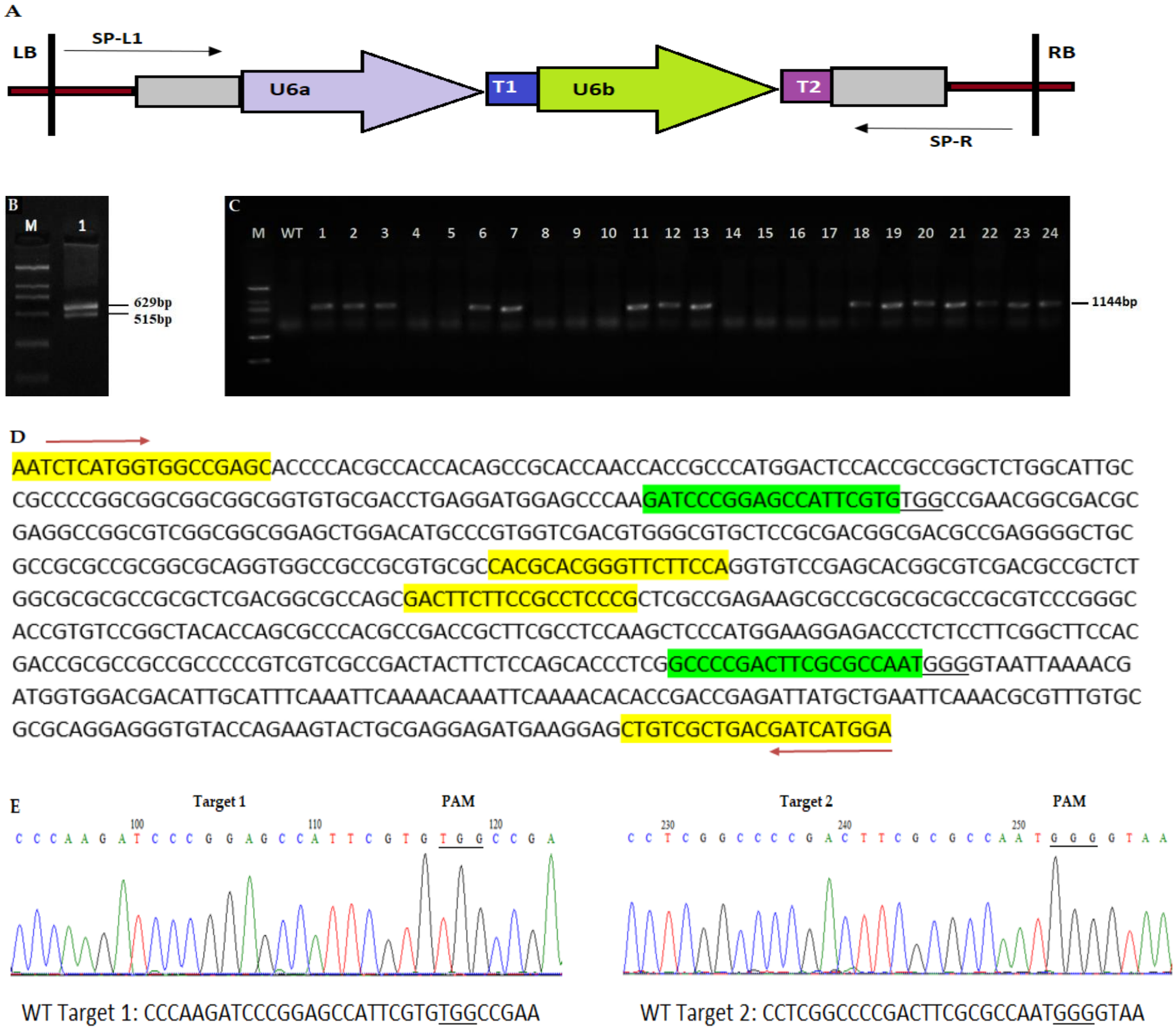


**Figure S8.** Detection and amplification of CRISPR/Cas9 T-DNA integration and the *OsGA20ox2* target sequence assembly in vector (**A**) Linking sequence of two expression cassettes U6a-driven Target 1 and U6b-driven Target 2 on pYL CRISPR/Cas9 vector (**B**) Gel electrophoresis detection of expression cassette for both targets, M: DL2000 DNA marker; (**C**) detection of expression cassettes after transformation of DH5$\alpha$; M: DL2000 DNA marker; 1~12: amplified bacterial colonies; 1144 bp: total amplified length of U6a and U6b expression cassette assembly; (**D**) Schematic representation of *SD1* target region showing the location and corresponding sequences of the two 20 bp gRNAs, gRNA1 and gRNA2 are highlighted in green while the PAM is underlined. Positions of forward and reverse primers flanking the target region are highlighted in yellow and indicated with red arrows, respectively; (**E**) Sequencing peak map of both target sites assembled in pYLCRISPR/Cas9 Pubi-*H* vector.
